# Optimisation of a triazolopyridine based histone demethylase inhibitor yields a potent and selective KDM2A (FBXL11) inhibitor†

**DOI:** 10.1039/C4MD00291A

**Published:** 2014-12-01

**Authors:** Katherine S. England, Anthony Tumber, Tobias Krojer, Giuseppe Scozzafava, Stanley S. Ng, Michelle Daniel, Aleksandra Szykowska, KaHing Che, Frank von Delft, Nicola A. Burgess-Brown, Akane Kawamura, Christopher J. Schofield, Paul E. Brennan

**Affiliations:** a Structural Genomics Consortium, University of Oxford, Old Road Campus, Roosevelt Drive, Headington OX3 7DQ, UK; b Chemistry Research Laboratory, University of Oxford, Mansfield Road, Oxford, OX1 3TA, UK; c Target Discovery Institute, University of Oxford, NDM Research Building, Roosevelt Drive, Oxford, OX3 7FZ, UK; d Diamond Light Source Ltd, Harwell Science and Innovation Campus, Didcot, OX11 0QX, UK; e Division of Cardiovascular Medicine, Radcliffe Department of Medicine, University of Oxford, Wellcome Trust Centre for Human Genetics, Roosevelt Drive, Oxford OX3 7BN, UK

## Abstract

A potent inhibitor of the JmjC histone lysine demethylase KDM2A (compound **35**, pIC_50_ 7.2) with excellent selectivity over representatives from other KDM subfamilies has been developed; the discovery that a triazolopyridine compound binds to the active site of JmjC KDMs was followed by optimisation of the triazole substituent for KDM2A inhibition and selectivity.

## Introduction

The dynamic methylation of histone lysine residues is an important process in transcriptional regulation. The introduction of *N*^ε^-methyl lysine methylation marks is catalysed by histone methyl transferases and their removal is catalysed by histone lysine demethylases (KDMs). Aberrant histone lysine methylation is associated with a variety of disease states, including cancer and ageing.^1,2^ Human KDMs are classified into two families according to their mechanisms; the lysine specific KDMs (KDM1A and B) which employ flavin adenine dinucleotide (FAD) as a cofactor and the 2-oxoglutarate (2OG) dependent KDMs, the JumonjiC (JmjC) KDMs,^3^ which are part of the wider 2OG oxygenase superfamily and which use 2OG and molecular oxygen as cofactors.^4,5^

The JmjC KDMs are grouped into five subfamilies (KDM2/7, KDM3, KDM4, KDM5 and KDM6).^6^ They have conserved 2OG and Fe(ii) binding sites, the precise nature of which are subfamily specific.^7^ The different members of the JmjC KDM family accept different substrates (typically methylated lysine residues on the *N*-terminal tails of histone H3, H4 and to a lesser extent H1);^8^ selectivity is mainly engendered through differences in the substrate binding pockets and by the presence of other recognition domains in addition to the JmjC domain.

KDM2A (FBXL11) is a member of the KDM2/7 subfamily, human members of which include KDM2B, PHF8 and KDM7A. KDM2A demethylates histone H3 residues, *i.e. N*^ε^-mono- and dimethyllysine-36 (H3K36me1/2),^9^ and has a role in cellular differentiation,^10^ regulation of NF-κB^11^ and cell proliferation.^12,13^ KDM2A is overexpressed in some non-small cell lung cancers (NSCLCs); knockdown of KDM2A with siRNA has been shown to reduce the proliferation of KDM2A-overexpressing NSCLC cell-lines indicating that KDM2A activity may promote proliferation of NSCLCs.^13^

**Fig. 1:**
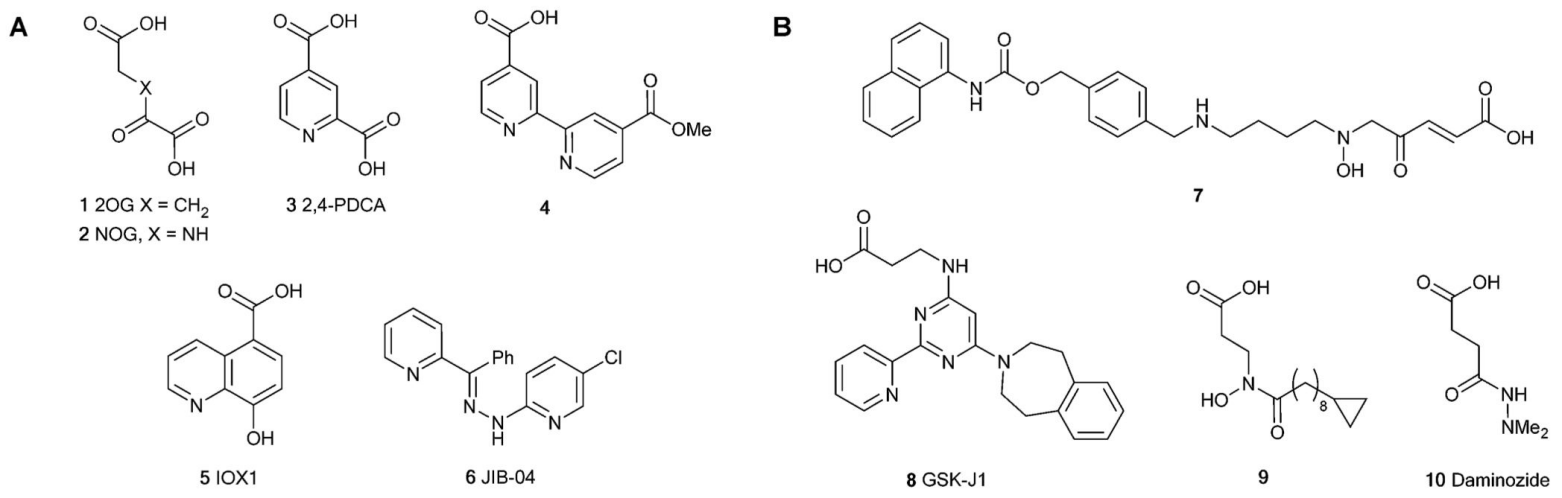
Structures of A. 2OG and broad spectrum JmjC KDM inhibitors (**1**–**6**) and B. Inhibitors with some selectivity for JmjC KDM subfamilies (**7**–**10**).

JmjC KDM inhibitors of varying selectivity have been described (Fig. 1A and Table 1; compounds **2**–**6**).^14-16^ Compounds with some degree of selectivity for the KDM2, KDM3 and KDM4 subfamilies have been discovered (Fig. 1B and Table 1); notably compound **7** which is approximately ten-fold selective for the KDM4 subfamily,^17^ compound **8** (GSK-J1) which is a KDM6A/B/C inhibitor,^18,19^ and the KDM2/7 inhibitor **9** (KDM2A pIC_50_ 5.8).^20^ Daminozide **10**, an agrochemical used to regulate plant growth, is the most potent KDM2/7 subfamily selective inhibitor reported in the literature to date (KDM2A pIC_50_ 5.8).^21^

**Fig. 2:**
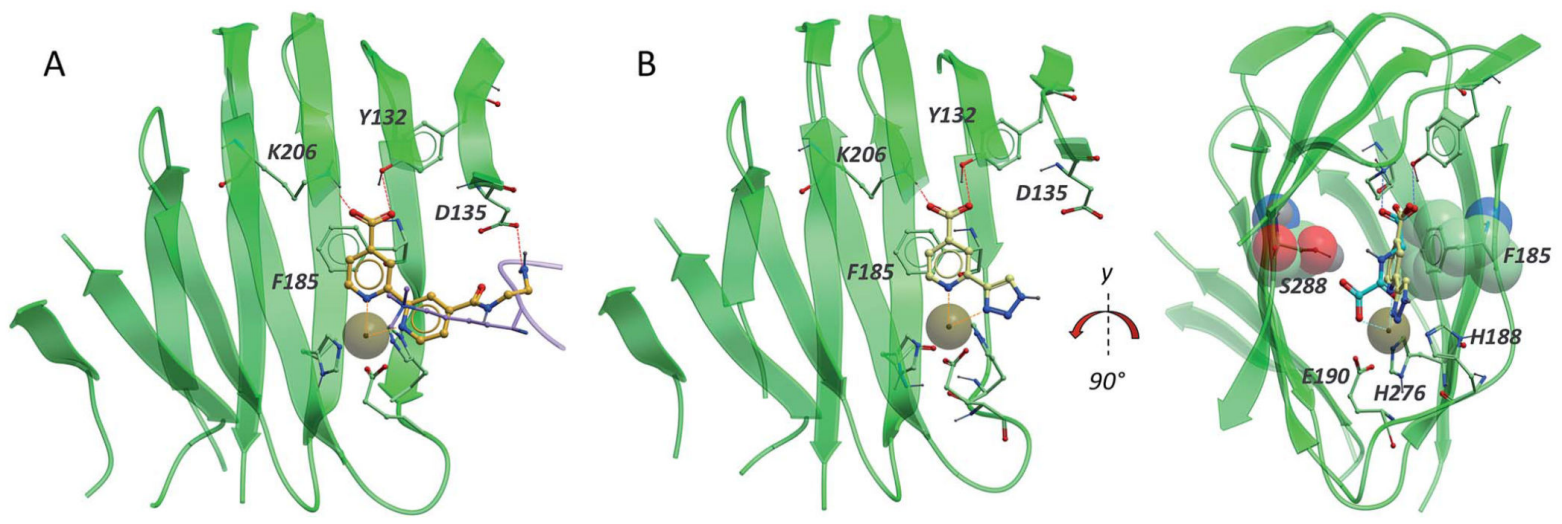
A. Overlay of a 2,2′-bipyridine inhibitor (orange sticks, from PDB ID: 3PDQ)^23^ and H3K9me3 peptide (lavender ribbon and sticks, from PDB ID: 2OQ6)^32^ in KDM4A (green ribbon and sticks, from PDB ID: 3PDQ). The inhibitor forms a H-bond to Y132 and two salt bridges to K206 and D135 (red dashed lines). The bipyridine motif coordinates the nickel atom (orange dashed lines to brown sphere) which replaces the catalytic iron in the crystal structure. The diaminoethane substituent extends into the peptide-binding pocket. B. Overlay of compound **14a** (pale sticks, from PDB ID: 4URA) and NOG, **2** (cyan sticks, from PDB ID: 2OQ6) in KDM4A (green ribbon and sticks, from PDB ID: 4URA). The catalytic iron, substituted by nickel in the crystal structure (brown sphere) is coordinated by the side chains of E190, H188 and H276. NOG, a close analogue of 2OG, forms one H-bond and one salt bridge to Y132 and K206 respectively (dark blue dashed lines) and bidentate coordination with the metal (light blue dashed lines). Triazole **14a** forms similar interactions with the catalytic metal (orange dashed lines), K206 and Y132 (red dashed lines) and is further stabilised by apparent aromatic stacking between the pyridine ring and F185 and van der Waals interactions with S288 (green stick and CPK).* Only the beta strand core of the protein is shown for clarity.

Most inhibitors of the JmjC KDMs described to date are 2OG mimetics,^7^ as indeed for 2OG oxygenases in general.^22^ For example a series of inhibitors has been developed based on the 2,2′-bipyridine scaffold where one of the pyridine rings bears a carboxylate group at the 4-position. X-ray crystallography of a derivative of compound **4** in complex with KDM4A demonstrates that the pyridine-N atoms bind to Fe(ii) in a bidentate manner and the 4-carboxy group mimics that of the 5-carboxy group of 2OG (Fig. 2A).^23^

Thus there is a need for more selective and/or potent inhibitors of JmjC KDMs in order to elucidate their physiological roles in healthy and diseased organisms and as starting points for medicinal chemistry programmes. Here we report the development of a highly selective and potent inhibitor of the JmjC KDM KDM2A.

## Results and discussion

In order to find selective inhibitors of JmjC KDMs, a new scaffold was designed based on the 2,2′-bipyridine series wherein one of the pyridine rings was replaced with a triazole ring (*e.g.* compound **14a**) to give a different potential iron binding motif. It was proposed that the triazole-N atom would be able to coordinate the catalytic iron atom and readily enable alternative vectors for compound elaboration. We envisaged that the planned route to the triazole series would enable synthesis of a range of analogues through construction of the triazole ring *via* copper-catalysed click reactions.^24,25^ This would allow rapid exploration of the structure activity relationship with the aim of exploiting differences in the substrate scope of the different JmjC KDM family members and achieving subfamily selectivity.

**Scheme 1:**
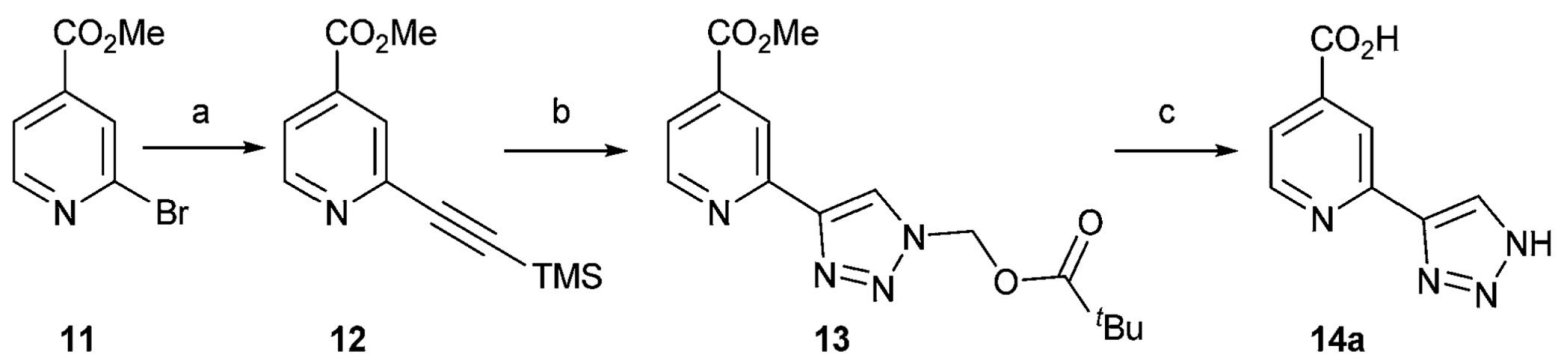
Synthesis of compound **14a**. Reagents and conditions: (a) TMSCCH, CuI, Pd(PPh_3_)_4_, Et_3_N, THF, 85%; (b) azidomethyl pivalate, TBAF, CuSO_4_·5H_2_O, (+)-sodium l-ascorbate, DMF, H_2_O, 65 °C, 53%; (c) NaOH, H_2_O, MeOH, 61%.

Methyl 2-[(trimethylsilyl)ethynyl]isonicotinate **12**^26^ was prepared from the corresponding bromide *via* Sonogashira coupling with trimethylsilylacetylene (TMSCCH) to give a protected alkyne intermediate as a precursor for copper-catalysed click reactions (Scheme 1).

The reaction between azidomethyl pivalate and the trimethylsilyl protected alkyne intermediate **12**, which was deprotected *in situ* with tetrabutylammonium fluoride (TBAF), gave pivaloyloxymethyl protected triazole **13** in a method modified from that described by Loren *et al.*^27^ Global deprotection using sodium hydroxide gave the N–H triazole target **14a**.

Compound **14a** was then screened against a panel of JmjC KDMs using AlphaScreen technology.^28^ The activity of compound **14a** was compared with that of the commercially available unsubstituted bipyridine analogue **15** in the same screening panel. Replacing the pyridine ring with the 1,2,3-triazole ring resulted in a greater than ten-fold reduction in potency for KDM3A, 4C, 4E, 5C and 6B, but a less than two-fold reduction in potency for KDM2A and 4A (Table 2). Although compound **14a** is very small (MW 190.2) it shows a preference for KDM2A and 4A/C. This is likely to be due to residue differences in the active site of the different JmjC KDMs.^4^ We therefore considered that the triazolopyridine scaffold may represent a good hit for the development of a selective KDM2A inhibitor.

Although it was not possible to crystallise compound **14a** with KDM2A it was co-crystallised with KDM4A as a surrogate. KDM2A and KDM4A have similar substrates and are both able to demethylate H3K36me2.^8^ The co-crystal structure of compound **14a** confirmed that a representative of the triazolopyridine series occupies the 2OG binding site in KDM4A (Fig. 2B) and provided insights to inform the design of KDM2A selective inhibitors.

**Scheme 2:**
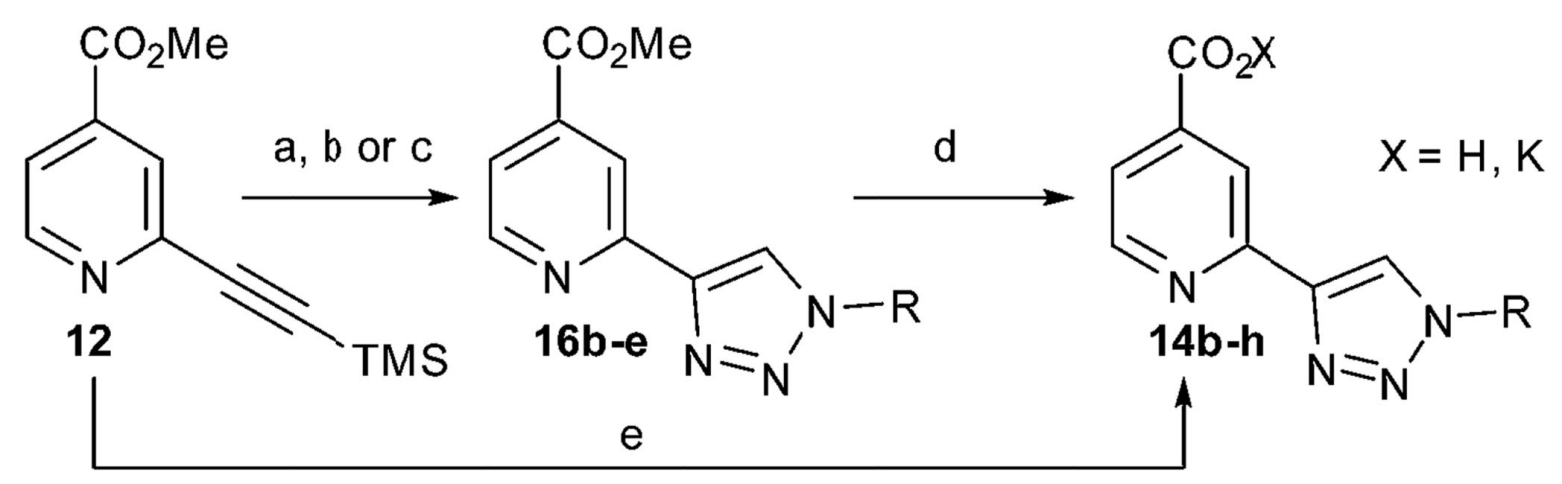
Synthesis of N-substituted triazoles. Reagents and conditions: (a) TBAF, BnN_3_, DIPEA, CuI, MeOH, 69%; (b) TBAF, RI, NaN_3_, CuSO_4_·5H_2_O, (+)-sodium l-ascorbate, DMF, H_2_O, 65 °C, 20–77%; (c) (i) KOTMS, MeCN, 81%; (ii) MeI, NaN_3_, CuSO_4_·5H_2_O, (+)-sodium l-ascorbate, DMF, H_2_O, 65 °C, 24%; (d) KOTMS, MeCN, 59–100%; (e) (i) TBAF, RN_3_, DIPEA, CuI, MeOH; (ii) KOTMS, MeCN, 14–50%.

The triazolopyridine scaffold itself does not extend substantially into the substrate pocket. However we anticipated that through the introduction of appropriate substituents on the triazole ring at N-1 and C-5, the activity and selectivity for KDM2A could be improved by making interactions in the substrate pocket. A diverse range of N-substituted triazole derivatives were synthesised from the trimethylsilyl protected alkyne intermediate **12** in a click triazole forming reaction either directly with functionalised azides or by generating substituted azides *in situ* in a one pot reaction between sodium azide and the corresponding alkyl or aryl iodide (Scheme 2).^29^

In addition to preparing triazole derivatives with simple alkyl substituents (Me, **14b** and Et, **14c**), derivatives bearing more complex substituents were also selected for synthesis. Substituents were selected so that the targets would have lead-like properties (*c*log*P* < 3, MW 200–350 g mol^−1^)^30^ and be amenable to rapid follow-up from suitable late-stage intermediates. For example benzyl and phenethyl substituents were selected with the view of synthesising substituted aryl systems from aryl halides. The piperidinyl derivative was selected to enable synthesis of substituted piperidines from the corresponding NH piperidine. Potential targets were docked into a KDM2A structure (PDB ID: 2YU1)^31^ based on the binding pose of compound **14a** in KDM4A. Compounds were selected for synthesis that could be accommodated in the enzyme pocket.

**Scheme 3:**
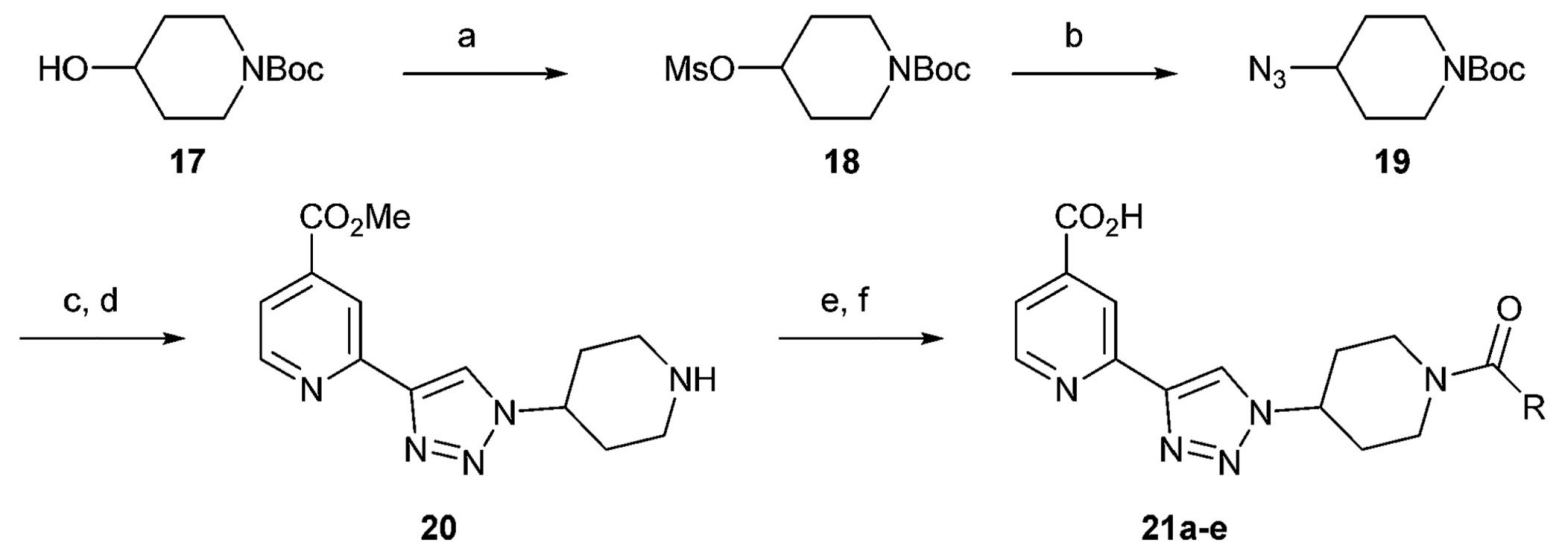
Synthesis of 4-piperidine derivatives. Reagents and conditions: (a) MsCl, Et_3_N, DCM, 59%; (b) NaN_3_, DMF, 60 °C, 65%; (c) **12**, TBAF, DIPEA, CuI, MeOH, 56%; (d) TFA, DCM, 73%; (e) RCOCl, Et_3_N, DCM; (f) LiOH (aq), MeOH.

When tested in the JmjC assay panel, these substituted triazoles maintained good selectivity for KDM2A over KDM4E and KDM6B; however, some of the compounds were not selective for KDM2A over KDM4A/C and KDM5C (Table 3). For example the methyl derivative **14b** is *ca.* three-fold more potent against KDM5C than KDM2A and the ethyl derivative **14c** is *ca.* five-fold more potent against KDM4C and 5C than KDM2A. The ethyl carbamate **14h** was selected as an attractive lead for substitution on the piperidine-N atom with the aim of improving selectivity through exploiting differences between the substrate binding pockets of KDM2A and KDM4A/C and 5C. *tert*-Butyloxycarbonyl (Boc)-protected 4-hydroxypiperidine was transformed to the 4-azido derivative *via* the methanesulfonate, then taken into the click reaction with the protected alkyne (Scheme 3). The Boc group was removed with CF_3_CO_2_H (TFA) to give a late-stage intermediate for functionalization with acid chlorides followed by pyridine ester deprotection.

The ethyl, *n*-propyl and phenyl substituted amides (**21a**–**c**) retained moderate activity against KDM2A (Table 4). Gratifyingly, the benzyl (**21d**) and phenylethyl (**21e**) substituents resulted in improved activity for KDM2A (pIC_50_ 5.4 and 5.6 respectively) and reduced activity for KDM3A (pIC_50_ 4.5 and <4.0) and KDM4A/C (pIC_50_ 4.1–4.5), however the 4-piperidine derivatives (**21a**–**e**) all inhibit KDM5C in the same range as KDM2A.

**Scheme 4:**
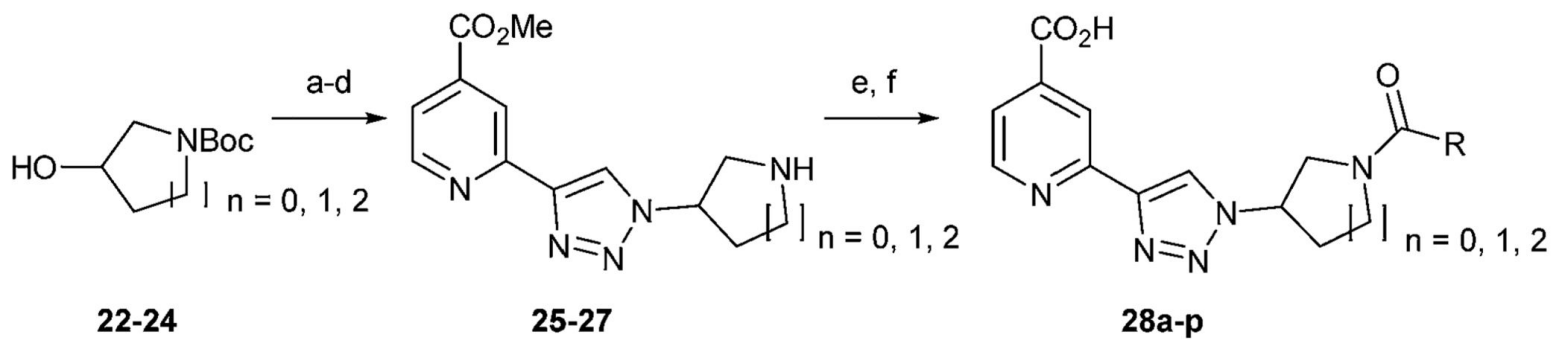
Synthesis of azetidine, pyrrolidine and 3-piperidine derivatives. Reagents and conditions: (a) MsCl, Et_3_N, DCM; (b) NaN_3_, DMF, 60 °C; (c) **12**, TBAF, DIPEA, CuI, MeOH; (d) TFA, DCM; (e) RCOCl, Et_3_N, DCM; (f) LiOH (aq), MeOH.

Given the differences in substrate selectivity of the KDM5 subfamily (H3K4me1/2/3) and the KDM2 subfamily (H3K36me1/2),^8^ we reasoned that selectivity for KDM2A over KDM5C could be achieved through further exploration of the histone substrate pocket. Other saturated heterocyclic ring systems were thus synthesised as alternatives to the 4-piperidine ring to explore different vectors within the pocket (Scheme 4). The chiral 3-piperidine and 3-pyrrolidine derivatives were prepared as racemates for initial screening.

The acetyl substituted azetidine **28a** manifested sub-micromolar KDM5C activity with greater than 85-fold selectivity over KDM2A, 3A, and 6B and three to seven-fold selectivity over the KDM4 subfamily. Potency against KDM5C was observed to decrease with increasing size of amide substituent on the amide (Table 5; compounds **28b**–**e**). KDM2A activity increased for the phenyl, benzyl and phenethyl-amides **28d**–**f**, but these still suffered from a lack of selectivity over KDM5C. The 3-substituted pyrrolidine compounds **28g**–**j** generally showed increased potency for KDM2A and improved selectivity over the other KDM representatives. The 3-piperidine series resulted in a dramatic increase in KDM2A inhibition with concurrent reduction in KDM5C activity. Excitingly, the most potent KMD2A inhibitor, the benzoyl 3-piperidine derivative **28n** (pIC_50_ 6.9) demonstrated greater than 50-fold selectivity for KDM2A over all six other JmjC KDMs in our panel.

**Scheme 5:**
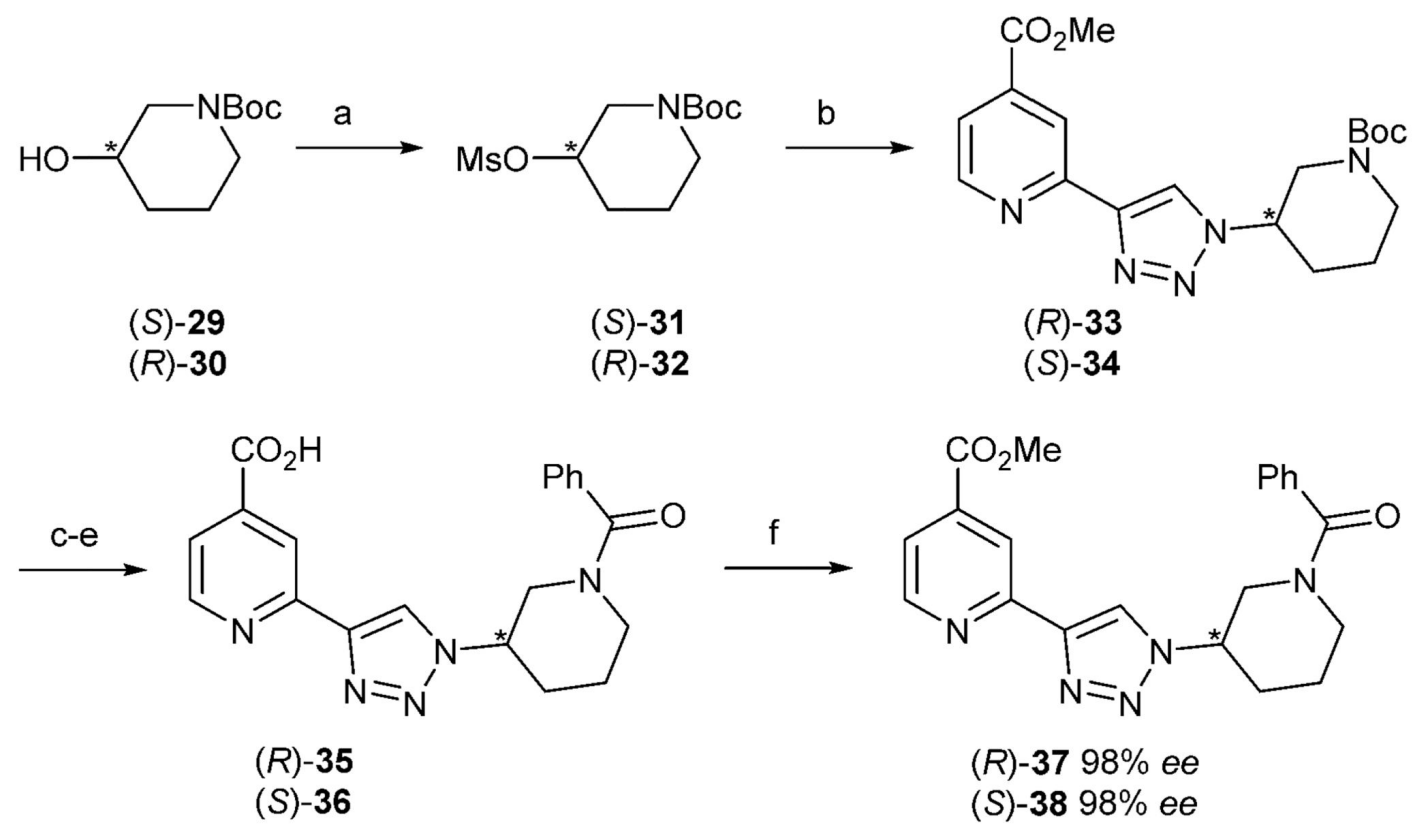
Synthesis of the (*R*)- and (*S*)-enantiomers of compound **28n** and conversion to the methyl ester derivatives. Reagents and conditions: for the (*R*)-enantiomer: (a) *tert*-butyl (3*S*)-3-hydroxypiperidine-1-carboxylate, MsCl, Et_3_N EtOAc, 96%; (b) (i) NaN_3_, DMF, 75 °C; (ii) **12**, TBAF, DIPEA, CuI, DMF, 48%; (c) TFA, DCM, 97%; (d) PhCOCl, Et_3_N, MeCN, quant.; (e) LiOH (aq), MeCN, 85%; (f) MeI, NaHCO_3_, DMF quant. For the (*S*)-enantiomer: (a) *tert*-butyl (3*R*)-3-hydroxypiperidine-1-carboxylate, MsCl, Et_3_N EtOAc, 98%; (b) (i) NaN_3_, DMF, 75 °C; (ii) **12**, TBAF, DIPEA, CuI, DMF, 34%; (c) TFA, DCM; (d) PhCOCl, Et_3_N, MeCN; (e) LiOH (aq), MeCN, 81% over 3 steps; (f) MeI, NaHCO_3_, DMF, 65%.

In order to investigate if the activity of compound **28n** is due to a single stereoisomer, the two enantiomers were synthesised from stereoisomerically pure Boc-protected alcohols which were converted to the corresponding azides. Click triazole formation, Boc deprotection, amide formation and ester hydrolysis gave enantiomers **35** and **36**. In order to verify the stereochemical integrity of compounds **35** and **36**, they were reconverted to the methyl ester derivatives **37** and **38** with MeI and NaHCO_3_ to enable analysis by chiral HPLC. The enantiomeric excesses were determined as 98%, confirming that racemisation had not occurred to any significant degree during the synthesis (Scheme 5).§

Gratifyingly, the (*R*)-enantiomer (**35**) is approximately 50-fold more potent than the (*S*)-enantiomer (**36**) indicating that the stereochemistry of the substituted piperidine plays an important role in facilitating binding to KDM2A and that the (*R*)-enantiomer positions the amide substituent in a favoured position for binding to KDM2A (Table 6). KDM4A/C and 5C activity also increased for the (*R*)-enantiomer relative to the racemic mixture, but inhibitor **35** is still greater than 100-fold selective for KDM2A over KDM4A/C/E, 30-fold selective over KDM5C and exhibits negligible activity on KDM3A and 6B at 100 μM.

Its potency and selectivity means that inhibitor **35** will be of interest as a tool molecule for the study of KDM2A in biological systems. The physico-chemical properties of compound **35** fall within the range predicted to give oral bioavailability by the Lipinski Rule of Five (MW < 500, log *P* < 5, H bond donors <5, H bond acceptors <10, Table 7).^35^ The KDM6A/B/C inhibitor **8** also complies with the Rule of Five but cellular activity has only been observed when it is dosed as the ethyl ester pro-drug,^18^ presumably due to poor membrane permeability as a result of deprotonation of the carboxylic acid at physiological pH (calculated p*K*_a_ 4.5). Compound **35** would also be expected to have poor membrane permeability as it is predicted to be more acidic than compound **8**. The cellular permeability of compound **35** and its methyl ester **37** was assessed using a parallel artificial membrane permeation assay (PAMPA, Table 7).^36^ The membrane permeability of compound **35** is predicted to be poor (0.41–1.9 × 10^−6^ cm s^−1^) however the methyl ester **37** is predicted to have good permeability (45–56 × 10^−6^ cm s^−1^). Biological investigations into the effects of inhibition of the KDM2/7 subfamily JmjC KDMs by both compound **35** and its methyl ester pro-drug **37** are currently underway.

## Conclusions

A new KDM inhibitor scaffold has been discovered through the incorporation of an alternative triazole metal binding motif to the known 2,2′-bipyridine-4-carboxylate scaffold. A co-crystal structure of the simplest example **14a** with KDM4A demonstrated that it binds to the JmjC KDMs *via* active site metal chelation. A number of analogues were synthesised leading to selective KDM inhibitors; the azetidine and piperidine substituted triazoles showed promise as selective KDM5C (*e.g.* compound 28b) and KDM2A (*e.g.* compound **28n**) inhibitors respectively. When prepared as a single enantiomer, compound **35** is a potent and selective inhibitor of KDM2A. Due to the similarity in the catalytic domain of the KDM2 and KDM7 subfamilies, it is expected that compound **35** will be a potent inhibitor of all members of these subfamilies. Compound **35** is significantly more potent than other KDM2/7 subfamily selective inhibitors reported in the literature to date, hydroxamate **9** and daminozide **10**.

## Supplementary Material

ESI

## Figures and Tables

**Table 1:** Inhibitory effect of some non-selective JmjC inhibitors (**2**–**6**) and inhibitors that are more selective for some JmjC subfamilies (**7**–**10**)

	pIC_50_^a^ KDM						
	2A	3A	4C	4E	5A	5C	6B
**2** ^14^	4.3	5.7		<4.0		5.1	6.5
**3** ^14^	5.4	5.1	5.6	5.2–6.0		6.3	4.5
**4** ^15^	5.3	6.0	5.5	5.3		7.5	5.0
**5** ^14^	4.8	6.8	6.2	6.5		4.6	7.0
**6** ^16^			6.0	6.5	6.6		6.1
**7** ^17^			5.5	5.2			4.4
**8** ^18,19^		4.4		4.7			7.2
**9** ^20^	5.2		4.1			4.3	
**10** ^21^	5.8	<4.0		<4.0		<4.0	<4.0

a Reported values determined in amplified luminescent proximity homogeneous assay (AlphaScreen), enzyme-linked immunosorbent assay (ELISA), dissociation-enhanced lanthanide fluorescent immunoassay (DELFIA) or matrix-assisted laser desorption/ionization (MALDI) assay.

**Table 2:** Inhibitory effect of compounds **14a** and **15** against seven JmjC KDMs

	pIC_50_^a^ KDM						
Structure	2A	3A	4A	4C	4E	5C	6B
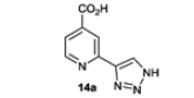	5.3 ± 0.20 (8)	4.7 ± 0.15 (2)	5.5 ± 0.33 (2)	5.2 ± 0.15 (4)	4.8 ± 0.15 (4)	4.9 ± 0.22 (4)	<4.0 (6)
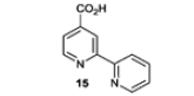	5.4 ± 0.17 (2)	5.9 ± 0.13 (4)	5.9 ± 0.07 (4)	6.7 ± 0.14 (2)	6.4 ± 0.19 (2)	6.2 ± 0.07 (2)	5.0 ± 0.13 (6)

a Mean pIC_50_ ± standard error of the mean (number of determinations) as determined by AlphaScreen.

**Table 3:** Inhibitory effect of N-substituted 1,2,3-triazole isonicotinic acids in seven JmjC KDMs

Compound		pIC_50_^a^ KDM						
14	R	2A	3A	4A	4C	4E	5C	6B
**14a**	H	5.3 ± 0.20 (8)	4.7 ± 0.15 (2)	5.5 ± 0.33 (2)	5.2 ± 0.14 (4)	4.8 ± 0.15 (4)	4.9 ± 0.22 (4)	<4.0 (6)
**14b**	Me	4.9 ± 0.18 (8)	4.6 ± 0.24 (2)	4.7 ± 0.19 (4)	5.0 ± 0.23 (4)	4.1 ± 0.10 (4)	5.2 ± 0.13 (4)	<4.0 (6)
**14c**	Et	4.5 ± 0.13 (8)	5.1 ± 0.21 (2)	ND	5.2 ± 0.19 (4)	4.1 ± 0.10 (2)	5.0 ± 0.10 (4)	<4.0 (4)
**14d**	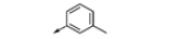	5.2 ± 0.16 (8)	5.1 ± 0.28 (2)	ND	5.3 ± 0.20 (4)	4.3 ± 0.21 (2)	5.3 ± 0.09 (4)	4.3 ± 0.17 (6)
**14e**	Bn	5.0 ± 0.14 (2)	4.9 ± 0.25 (2)	4.1 ± 0.11 (4)	4.4 ± 0.12 (2)	<4.0 (4)	5.1 ± 0.13 (2)	<4.0 (4)
**14f**	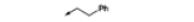	5.1 ± 0.12 (2)	5.5 ± 0.13 (2)	4.2 ± 0.12 (4)	4.4 ± 0.08 (2)	4.0 ± 0.20 (2)	5.1 ± 0.16 (2)	4.1 ± 0.17 (4)
**14g**	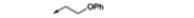	5.2 ± 0.10 (2)	5.0 ± 0.19 (2)	<4.0 (2)	<4.0 (2)	<4.0 (2)	5.0 ± 0.09 (2)	4.1 ± 0.12 (4)
**14h**	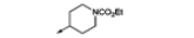	5.1 ± 0.17 (2)	4.7 ± 0.28 (2)	<4.0 (2)	<4.0 (2)	<4.0 (2)	5.4 ± 0.09 (2)	<4.0 (4)

a Mean pIC_50_ ± standard error of the mean (number of determinations) as determined by AlphaScreen.

**Table 4:** Inhibitory effect of the substituted 4-piperidine series in seven JmjC KDMs

Compound		pIC_50_^a^ KDM						
21	R	2A	3A	4A	4C	4E	5C	6B
**21a**	Et	5.2 ± 0.14 (2)	4.3 ± 0.13 (2)	ND	4.8 ± 0.42 (2)	<4.0 (2)	5.3 ± 0.11 (2)	4.2 ± 0.11 (2)
**21b**	^*n*^Pr	5.1 ± 0.14 (2)	<4.0 (2)	ND	4.8 ± 0.19 (2)	<4.0 (2)	5.2 ± 0.07 (2)	<4.0 (2)
**21c**	Ph	5.1 ± 0.13 (2)	<4.0 (2)	4.3 ± 0.29 (4)	5.0 ± 0.18 (4)	<4.0 (2)	5.4 ± 0.02 (2)	4.4 ± 0.26 (2)
**21d**	Bn	5.4 ± 0.11 (2)	4.5 ± 0.19 (2)	4.4 ± 0.30 (2)	4.5 ± 0.19 (2)	<4.0 (2)	5.4 ± 0.06 (2)	4.8 ± 0.09 (2)
**21e**	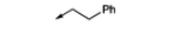	5.6 ± 0.09 (4)	<4.0 (2)	4.1 ± 0.18 (2)	4.1 ± 0.15 (2)	<4.0 (2)	5.5 ± 0.06 (2)	<4.0 (2)

a Mean pIC_50_ ± standard error of the mean (number of determinations) as determined by AlphaScreen.

**Table 5:** Inhibitory effect of azetidine, pyrrolidine and 3-piperidine derivatives in seven JmjC KDMs

Compound			pIC_50_^a^ KDM						
28	*n*	R	2A	3A	4A	4C	4E	5C	6B
**28a**	0	Me	<4.0 (2)	4.3 ± 0.17 (2)	5.6 ± 0.29 (2)	5.8 ± 0.19 (2)	5.3 ± 0.07 (2)	6.2 ± 0.07 (2)	<4.0 (2)
**28b**		Et	4.1 ± 0.15 (2)	<4.0 (2)	5.3 ± 0.22 (2)	4.9 ± 0.31 (2)	<4.0 (2)	5.6 ± 0.04 (2)	4.3 ± 0.87 (2)
**28c**		^*n*^Pr	4.3 ± 0.13 (2)	<4.0 (2)	5.3 ± 0.26 (2)	5.1 ± 0.19 (2)	4.1 ± 0.24 (2)	5.2 ± 0.06 (2)	4.2 ± 0.83 (2)
**28d**		Ph	5.3 ± 0.21 (2)	4.5 ± 0.07 (2)	<4.0 (4)	4.7 ± 0.14 (2)	4.3 ± 0.07 (2)	5.4 ± 0.03 (2)	4.0 ± 0.03 (2)
**28e**		Bn	5.2 ± 0.20 (2)	4.0 ± 0.07 (2)	ND	4.4 ± 0.13 (4)	<4.0 (2)	5.2 ± 0.10 (2)	<4.0 (2)
**28f**		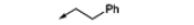	5.0 ± 0.20 (2)	<4.0 (2)	5.2 ± 0.25 (2)	5.1 ± 0.12 (4)	4.6 ± 0.11 (2)	5.9 ± 0.05 (2)	<4.0 (2)
**28g**	1	^*n*^Pr	4.9 ± 0.10 (2)	<4.0 (4)	ND	4.7 ± 0.15 (6)	<4.0 (4)	5.5 ± 0.13 (4)	<4.0 (4)
**28h**		Ph	6.4 ± 0.10 (2)	ND	ND	ND	ND	ND	ND
**28i**		Bn	6.0 ± 0.09 (2)	4.2 ± 0.19 (2)	ND	4.5 ± 0.18 (2)	<4.0 (2)	5.2 ± 0.06 (2)	4.3 ± 0.63 (2)
**28j**		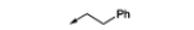	ND	4.0 ± 0.10 (2)	ND	4.4 ± 0.16 (2)	<4.0 (2)	5.7 ± 0.07 (2)	4.1 ± 0.03 (2)
**28k**	2	Me	4.9 ± 0.15 (2)	4.4 ± 0.27 (2)	4.4 ± 1.65 (2)	4.8 ± 0.21 (2)	<4.0 (2)	5.6 ± 0.06 (2)	4.0 ± 0.17 (2)
**28l**		Et	5.1 ± 0.17 (2)	4.2 ± 0.17 (2)	ND	4.8 ± 0.38 (2)	4.0 ± 0.13 (2)	5.4 ± 0.05 (2)	4.3 ± 0.16 (2)
**28m**		^*n*^Pr	5.3 ± 0.09 (2)	4.5 ± 0.22 (2)	ND	4.7 ± 0.29 (2)	4.0 ± 0.15 (2)	5.3 ± 0.06 (2)	4.1 ± 0.10 (2)
**28n**		Ph	6.9 ± 0.13 (6)	4.3 ± 0.10 (2)	4.2 ± 1.13 (2)	4.7 ± 0.14 (14)	<4.0 (10)	5.0 ± 0.11 (2)	4.7 ± 0.13 (8)
**28o**		Bn	5.7 ± 0.08 (2)	4.6 ± 0.14 (2)	ND	4.7 ± 0.42 (2)	<4.0 (2)	5.1 ± 0.27 (2)	4.9 ± 0.19 (2)
**28p**		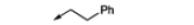	6.2 ± 0.08 (2)	4.3 ± 0.17 (2)	ND	4.6 ± 0.78 (4)	<4.0 (2)	5.3 ± 0.07 (2)	4.9 ± 0.13 (2)

a Mean pIC_50_ ± standard error of the mean (number of determinations) as determined by AlphaScreen.

**Table 6:** Inhibitory effect of the two enantiomers of **28n** in seven JmjC KDMs

	pIC_50_^a^ KDM						
Compound	2A	3A	4A	4C	4E	5C	6B
35	7.2 ± 0.16 (4)	<4.0 (4)	4.8 ± 0.28 (2)	4.8 ± 0.10 (4)	<4.0 (2)	5.7 ± 0.07 (2)	<4.0 (4)
36	5.5 ± 0.09 (2)	ND	ND	<4.0 (6)	<4.0 (6)	ND	4.5 ± 0.24 (2)

a Mean pIC_50_ ± standard error of the mean (number of determinations) as determined by AlphaScreen.

**Table 7:** Selected properties of compounds **8**, **35** and **37**

	Physicochemical properties						PAMPA *P*_e_ (10^−6^ cm s^−1^)		
Compound	MW	H bond donors	H bond acceptors	*c* log *P*^a^	*c* log *D* at pH 7.4^a^	Carboxylic acid *c*-p*K*_a_^a^	pH 7.4	pH 6.2	pH 5.0
8	389.5	2	7	3.8	1.2	4.2	ND	ND	ND
35	377.4	1	8	2.5	−1.3	3.0	0.69	0.41	1.9
37	391.4	0	8	2.6	2.6	N/A	52	45	56

a Properties calculated using ACD labs prediction model.^33,34^

## Footnotes

§ **35** and **36** were synthesised from stereoisomerically pure Boc- protected alcohols which were purchased from Sigma Aldrich Ltd. The alcohols were converted to the methanesulfonates and then reacted with sodium azide. In assigning the stereochemistry of **35** and **36** we have assumed that displacement with sodium azide occurred with inversion of configuration.

## References

[1] Arrowsmith CH, Bountra C, Fish PV, Lee K, Schapira M (2012). Nat. Rev. Drug Discovery.

[2] Greer EL, Shi Y (2012). Nat. Rev. Genet.

[3] Kooistra SM, Helin K (2012). Nat. Rev. Mol. Cell Biol.

[4] McDonough MA, Loenarz C, Chowdhury R, Clifton IJ, Schofield CJ (2010). Curr. Opin. Struct. Biol.

[5] Aik W, McDonough MA, Thalhammer A, Chowdhury R, Schofield CJ (2012). Curr. Opin. Struct. Biol.

[6] Klose RJ, Kallin EM, Zhang Y (2006). Nat. Rev. Genet.

[7] Thinnes CC, England KS, Kawamura A, Chowdhury R, Schofield CJ, Hopkinson RJ (2014). Biochim. Biophys. Acta, Gene Regul. Mech.

[8] Walport LJ, Hopkinson RJ, Schofield CJ (2012). Curr. Opin. Chem. Biol.

[9] Tsukada Y, Fang J, Erdjument-Bromage H, Warren ME, Borchers CH, Tempst P, Zhang Y (2006). Nature.

[10] Du J, Ma Y, Ma P, Wang S, Fan Z (2013). Stem Cells.

[11] Lu T, Jackson MW, Wang B, Yang M, Chance MR, Miyagi M, Gudkov AV, Stark GR (2010). Proc. Natl. Acad. Sci. U. S. A.

[12] Gao R, Dong R, Du J, Ma P, Wang S, Fan Z (2013). Mol. Cell. Biochem.

[13] Wagner KW, Alam H, Dhar SS, Giri U, Li N, Wei Y, Giri D, Cascone T, Kim J-H, Ye Y, Multani AS, Chan C-H, Erez B, Saigal B, Chung J, Lin H-K, Wu X, Hung M-C, Heymach JV, Lee MG (2013). J. Clin. Invest.

[14] Hopkinson RJ, Tumber A, Yapp C, Chowdhury R, Aik W, Che KH, Li XS, Kristensen JBL, King ONF, Chan MC, Yeoh KK, Choi H, Walport LJ, Thinnes CC, Bush JT, Lejeune C, Rydzik AM, Rose NR, Bagg EA, McDonough MA, Krojer TJ, Yue WW, Ng SS, Olsen L, Brennan PE, Oppermann U, Muller S, Klose RJ, Ratcliffe PJ, Schofield CJ, Kawamura A (2013). Chem. Sci.

[15] Rotili D, Tomassi S, Conte M, Benedetti R, Tortorici M, Ciossani G, Valente S, Marrocco B, Labella D, Novellino E, Mattevi A, Altucci L, Tumber A, Yapp C, King ONF, Hopkinson RJ, Kawamura A, Schofield CJ, Mai A (2013). J. Med. Chem.

[16] Wang L, Chang J, Varghese D, Dellinger M, Kumar S, Best AM, Ruiz J, Bruick R, Peña-Llopis S, Xu J, Babinski DJ, Frantz DE, Brekken RA, Quinn AM, Simeonov A, Easmon J, Martinez ED (2013). Nat. Commun.

[17] Luo X, Liu Y, Kubicek S, Myllyharju J, Tumber A, Ng S, Che KH, Podoll J, Heightman TD, Oppermann U, Schreiber SL, Wang X (2011). J. Am. Chem. Soc.

[18] Kruidenier L, Chung C.-w., Cheng Z, Liddle J, Che K, Joberty G, Bantscheff M, Bountra C, Bridges A, Diallo H, Eberhard D, Hutchinson S, Jones E, Katso R, Leveridge M, Mander PK, Mosley J, Ramirez-Molina C, Rowland P, Schofield CJ, Sheppard RJ, Smith JE, Swales C, Tanner R, Thomas P, Tumber A, Drewes G, Oppermann U, Patel DJ, Lee K, Wilson DM (2012). Nature.

[19] Walport LJ, Hopkinson RJ, Vollmar M, Madden SK, Gileadi C, Oppermann U, Schofield CJ, Johansson C (2014). J. Biol. Chem.

[20] Suzuki T, Ozasa H, Itoh Y, Zhan P, Sawada H, Mino K, Walport L, Ohkubo R, Kawamura A, Yonezawa M, Tsukada Y, Tumber A, Nakagawa H, Hasegawa M, Sasaki R, Mizukami T, Schofield CJ, Miyata N (2013). J. Med. Chem.

[21] Rose NR, Woon ECY, Tumber A, Walport LJ, Chowdhury R, Li XS, King ONF, Lejeune C, Ng SS, Krojer T, Chan MC, Rydzik AM, Hopkinson RJ, Che KH, Daniel M, Strain-Damerell C, Gileadi C, Kochan G, Leung IKH, Dunford J, Yeoh KK, Ratcliffe PJ, Burgess-Brown N, von DF, Muller S, Marsden B, Brennan PE, McDonough MA, Oppermann U, Klose RJ, Schofield CJ, Kawamura A (2012). J. Med. Chem.

[22] Rose NR, McDonough MA, King ONF, Kawamura A, Schofield CJ (2011). Chem. Soc. Rev.

[23] Chang K-H, King ONF, Tumber A, Woon ECY, Heightman TD, McDonough MA, Schofield CJ, Rose NR (2011). ChemMedChem.

[24] Rostovtsev VV, Green LG, Fokin VV, Sharpless KB (2002). Angew. Chem., Int. Ed.

[25] Tornøe CW, Christensen C, Meldal M (2002). J. Org. Chem.

[26] Bagley SW, Griffith DA, Kung DW-S (2011). WO Pat.

[27] Loren JC, Krasiśski A, Fokin VV, Sharpless KB (2005). Synlett.

[28] Kawamura A, Tumber A, Rose NR, King ONF, Daniel M, Oppermann U, Heightman TD, Schofield C (2010). Anal. Biochem.

[29] Thibault RJ, Takizawa K, Lowenheilm P, Helms B, Mynar JL, Fréchet JMJ, Hawker CJ (2006). J. Am. Chem. Soc.

[30] Nadin A, Hattotuwagama C, Churcher I (2012). Angew. Chem., Int. Ed.

[31] Han Z, Liu P, Gu L, Zhang Y, Li H, Chen S, Chai J (2007). Frontier Science.

[32] Ng SS, Kavanagh KL, McDonough MA, Butler D, Pilka ES, Lienard BMR, Bray JE, Savitsky P, Gileadi O, von Delft F, Rose NR, Offer J, Scheinost JC, Borowski T, Sundstrom M, Schofield CJ, Oppermann U (2007). Nature.

[33] Petrauskas A, Kolovanov E (2000). Perspect. Drug Discovery Des.

[34] Meloun M, Bordovská S (2007). Anal. Bioanal. Chem.

[35] Lipinski CA (2004). Drug Discovery Today: Technol.

[36] Avdeef A, Tsinman O (2006). Eur. J. Pharm. Sci.

