# Supplementary material for: Optimisation of a triazolopyridine based histone demethylase inhibitor yields a potent and selective KDM2A (FBXL11) inhibitor
Source: Medchemcomm. Author manuscript; Available in PMC 2015 Dec 15. (PMC4678576; doi:10.1039/C4MD00291A)
Supplement: ESI [file NIHMS66085-supplement-ESI.pdf]

Electronic Supplementary Material (ESI) for Medicinal Chemistry Communications.
This journal is © The Royal Society of Chemistry 2014

Supplementary Material for:

Optimisation of a Triazolopyridine Based Histone Demethylase Inhibitor Yields a Potent and Selective KDM2A (FBXL11) Inhibitor

Katherine S. England, Anthony Tumber, Tobias Krojer, Giuseppe Scozzafava, Stanley S. Ng, Michelle Daniel, Aleksandra Szykowska, KaHing Che, Frank von Delft, Nicola A. Burgess-Brown, Akane Kawamura, Christopher J. Schofield and Paul E. Brennan*

1. Synthesis of Representative Compounds

General Experimental

Commercial reagents and solvents were used without further purification. Water was purified using an Elix® UV-10 system. NMR spectra were recorded on a Bruker DPX200 (200 MHz), Bruker AV400 (400 MHz), Varian Mercury 400 MHz (400 MHz) or Bruker AVII 500 (500 MHz) in the deuterated solvent stated. Coupling constants (*J*) are expressed in Hz and are recorded to the nearest 0.5 Hz. The number of protons (n) for a given resonance signal is indicated by nH. Mass spectra were recorded using a Waters LCT Premier or Micromass Platform 1 spectrometer, operating in positive or negative modes, from solutions of methanol. *m/z* values are reported in Daltons and are followed by their percentage abundance in parentheses. High resolution mass spectra (HRMS) were recorded using a Bruker MicroTOF machine calibrated using polyalanine.

Analytical reverse-phase LCMS was used to determine the purity of the synthesized compounds using either a WATERS sunfire C18 column (system A; used unless otherwise stated), a Gemini-NX C18 110A column (system B), a WATERS sunfire C18 column (system C), an Xbridge C18 column (system D) or a Merk Millipore Chromolith Performance RP-18e column (system E). System A used electrospray ionization, operating in positive or negative mode; separation was achieved using a linear gradient of solvent A (water + 0.01% CF_3CO_2H) and solvent B (acetonitrile + 0.01% CF_3CO_2H), eluting at a flow rate of 1 mL/min and monitoring at 254 nm: 0% B over 2 min, 0% B to 100% B over 16 min and 100 % B over 2 min. System B used electrospray ionization, operating in positive mode or negative mode; separation was achieved using a linear gradient of solvent A (water + 0.1% HCO_2H) and solvent B (acetonitrile + 0.1% HCO_2H), eluting at a flow rate of 1.5 mL/min and monitoring

at 225 nm: 5% B to 95% B over 3 min and 95 % B over 1 min. System C used electrospray ionization, operating in positive or negative mode; separation was achieved using a linear gradient of solvent A (water + 0.01% CF_3CO_2H) and solvent B (acetonitrile + 0.01% CF_3CO_2H), eluting at a flow rate of 2.5 mL/min and monitoring at 210, 254 and 280 nm: 5% B to 95% B over 2 min and 95 % B over 1.5 min. System D used electrospray ionization, operating in positive mode; separation was achieved using a linear gradient of solvent A (water + 0.05% CF_3CO_2H) and solvent B (acetonitrile + 0.05% CF_3CO_2H), eluting at a flow rate of 1.8 mL/min and monitoring at 254 and 280 nm: 5% B to 95% B over 1.2 min and 95% B over 1.5 min. System E used electrospray ionization, operating in positive or negative mode; separation was achieved using a linear gradient of solvent A (water + 0.01% CF_3CO_2H) and solvent B (acetonitrile + 0.01% CF_3CO_2H), eluting at a flow rate of 1 mL/min and monitoring at 254 nm: 2% B over 2 min, 2% B to 100% B over 8 min and 100 % B over 1 min.

Enantiomeric excesses were determined by HPLC analysis employing a Chiralpak® AD analytical column. Separation was achieved using hexane:*iso*-propanol 80:20, eluting at a flow rate of 0.8 mL/min and monitoring at 254 nm and by comparing the samples to a 1:1 mixture of each enantiomer.

Elemental analyses were recorded by the elemental analysis service of the London Metropolitan University.

Synthesis of Representative Compounds

Compound **12** was synthesized according to a literature procedure and its physical and spectroscopic properties are in agreement to those reported.[1]

Methyl 2-[(trimethylsilyl)ethynyl]isonicotinate (12)

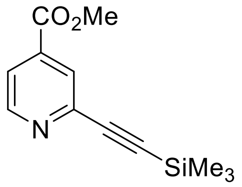

A solution of methyl 2-bromopyridine-4-carboxylate (5.40 g, 25.0 mmol), copper (I) iodide (571 mg, 3.00 mmol), tetrakis(triphenylphosphine)palladium (722 mg, 0.63 mmol) and

triethylamine (87.1 mL, 130 mmol) in tetrahydrofuran (43.5 mL) was evacuated and refilled with nitrogen (x 5), trimethylsilylacetylene (7.6 mL, 55.0 mmol) was then added and the resulting solution stirred at room temperature for 16 h. The reaction mixture was concentrated *in vacuo* to give a black gum that was taken up in dichloromethane (40 mL) and filtered. The filtrate was concentrated *in vacuo* and purified by flash column chromatography (9:1 heptane:ethyl acetate) to give **12** as an orange oil (4.94 g, 85%). R_f 0.20 (9:1 heptane:ethyl acetate); δ_H (400 MHz, $CDCl_3$) 0.26 (9H, s), 3.94 (3H, s), 7.75 (1H, dd, *J* 5.0, 2.0), 7.97 – 7.99 (1H, m), 8.70 (1H, dd, *J* 5.0, 1.0); *m/z* (MS, ES^+) 234 (100%, MH^+); LCMS tR 5.9 min (254 nm: 100%); accurate mass: found 234.0947, calc. for $C_{12}H_{16}NO_2Si^+$ 234.0945.

Compound **s1** was synthesized according to a literature procedure and its physical and spectroscopic properties are in agreement to those reported.[2]

Azidomethyl pivalate (s1)

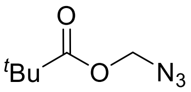

Chloromethyl pivalate (0.86 mL, 6.0 mmol) was added to a solution of sodium azide (429 mg, 6.6 mmol) in water (3.5 mL) and the resulting solution heated at 90 °C for 16 h. The solution was diluted with water (15 mL) then extracted with dichloromethane (2 x 20 mL). The organic layers were combined, dried over sodium sulfate and concentrated *in vacuo* to give **s1** as a colourless liquid (698 mg, 74%). δ_H (400 MHz, $CDCl_3$) 1.26 (9H, s, 3 x $C\underline{H}_3$), 5.14 (2H, s, $C\underline{H}_2$).

Methyl 2-(1-{[(2,2-dimethylpropanoyl)oxy]methyl}-1*H*-1,2,3-triazol-4-yl)isonicotinate (13)

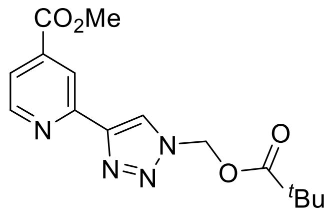

A solution of tetrabutylammonium fluoride in water (75% w/w, 85 µL, 0.24 mmol) was added dropwise over 5 min to a mixture of methyl 2-[(trimethylsilyl)ethynyl]isonicotinate (57 mg, 0.24 mmol), azidomethyl pivalate (42 mg, 0.27 mmol), cupric sulfate pentahydrate (3

mg, 0.01 mmol) and (+)-sodium L-ascorbate (5 mg, 0.03 mmol) in 1 mL of a 4:1 mixture of dimethylformamide and water. The resulting suspension was heated at 65 °C for 16 h. The reaction mixture was diluted with brine (15 mL) and extracted with ethyl acetate (2 x 20 mL). The organic layers were washed with brine (2 x 15 mL) then combined, dried over sodium sulfate and concentrated *in vacuo* to give a brown oil that was purified by flash column chromatography (10% → 30% ethyl acetate in cyclohexane) to give **13** as a white solid (41 mg, 53%). R_f 0.40 (2:1 cyclohexane:ethyl acetate); mp 78 – 81 °C; δ_H (400 MHz, $CDCl_3$) 1.20 (9H, s), 3.98 (3H, s), 6.32 (2H, s), 7.80 (1H, dd, *J* 5.0, 1.5), 8.42 (1H, s), 8.70 (1H, dd, *J* 1.5, 1.0), 8.74 (1H, dd, *J* 5.0, 1.0); *m/z* (MS, ES^+) 319 (100%, MH^+); LCMS tR 15.2 min (254 nm: 99%); accurate mass: found 341.1209, calc. for $C_{15}H_{18}N_4NaO_4^+$ 341.1220.

2-(1*H*-1,2,3-Triazol-4-yl)isonicotinic acid (14a)

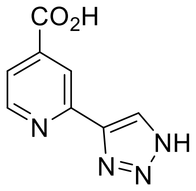

An aqueous solution of sodium hydroxide (1M, 0.6 mL, 0.6 mmol) was added to a solution of methyl 2-(1-{[(2,2-dimethylpropanoyl)oxy]methyl}-1*H*-1,2,3-triazol-4-yl)isonicotinate (80 mg, 0.25 mmol) in methanol (0.6 mL). The resulting suspension was stirred at room temperature for 16 h. The reaction mixture was concentrated *in vacuo*, diluted with water (10 mL), acidified to pH 1 with 1M aqueous hydrochloric acid and extracted with ethyl acetate (3 x 10 mL). The organic layers were combined, dried over sodium sulfate and concentrated *in vacuo* to give **14a** as a white solid (29 mg, 61%). mp 298 – 300 °C; δ_H (500 MHz, $(CD_3)_2SO$) 7.77 (1H, dd, *J* 5.0, 1.5), 8.39 (1H, s), 8.42 – 8.49 (1H, m), 8.80 (1H, d, *J* 5.0); *m/z* (MS, ES^-) 189 (100%, $[M-H]^-$); LCMS tR 8.2 min (254 nm: 100%); accurate mass: found 189.0421, calc. for $C_8H_5N_4O_2^-$ 189.0418.

Potassium 2-ethynylisonicotinate (s2)

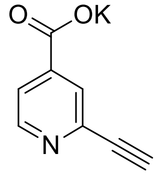

Potassium trimethylsilanolate (141 mg, 1.10 mmol) was added to a solution of methyl 2-[(trimethylsilyl)ethynyl]isonicotinate (233 mg, 1.00 mmol) in acetonitrile (40 mL) and the resulting suspension stirred at room temperature for 16 h. The resulting precipitate was filtered, washed with acetonitrile (2 x 5 mL) and dried under vacuum to give **s2** as a beige solid (150 mg, 81%). R_f 0.30 (4:1 dichloromethane:methanol); mp 370 – 372 °C; δ_H (400 MHz, $(CD_3)_2SO$) 4.24 (1H, s), 7.68 (1H, dd, *J* 5.0, 1.5), 7.81 (1H, app. s), 8.48 (1H, d, *J* 5.0); *m/z* (MS, ES^-) 146 (100%, $[M\text{-}K]^-$); LCMS tR 8.9 min (254 nm: 85%); accurate mass: found 146.0243, calc. for $C_8H_4NO_2^-$ 146.0248.

Methyl 2-(1-methyl-1*H*-1,2,3-triazol-4-yl)isonicotinate (16b)

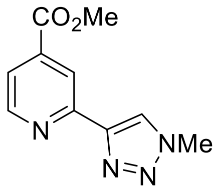

Methyl iodide (20.5 μL, 0.33 mmol) was added to a solution of potassium 2-ethynylisonicotinate (56 mg, 0.30 mmol), sodium azide (23 mg, 0.36 mmol), cupric sulfate pentahydrate (4 mg, 0.02 mmol) and (+)-sodium L-ascorbate (7 mg, 0.03 mmol) in 2 mL of a 4:1 mixture of dimethylformamide and water. The resulting solution was heated at 65 °C for 16 h. The reaction mixture was diluted with brine (10 mL) and extracted with ethyl acetate (2 x 10 mL). The organic layers were washed with brine (2 x 10 mL) then combined, dried over sodium sulfate and concentrated *in vacuo*. The residue was washed with hexane (10 mL) to give **16b** as a brown solid (16 mg, 24%). mp 108 – 110°C; δ_H (400 MHz, $CDCl_3$) 3.97 (3H, s), 4.18 (3H, s), 7.76 – 7.79 (1H, m), 8.15 (1H, s), 8.68 – 8.73 (2H, m); *m/z* (MS, ES^+) 219 (100%, MH^+); LCMS tR 10.7 min (254 nm: 88%); accurate mass: found 241.0692, calc. for $C_{10}H_{10}N_4NaO_2^+$ 241.0696.

Potassium 2-(1-methyl-1*H*-1,2,3-triazol-4-yl)isonicotinate (14b)

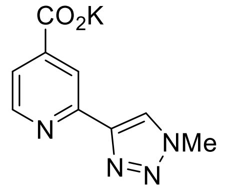

Methyl 2-(1-methyl-1*H*-1,2,3-triazol-4-yl)isonicotinate (12 mg, 0.06 mmol) was dissolved in acetonitrile (4 mL), potassium trimethylsilanolate (11 mg, 0.08 mmol) was added and the resulting suspension stirred at room temperature for 16 h, the volatiles removed *in vacuo*, the residue taken up in methanol (2 mL) then filtered and washed with methanol (2 x 2 mL). The filtrate was concentrated *in vacuo* to give an orange solid that was washed with acetonitrile (3 x 2 mL) then dried under vacuum to give **14b** as a yellow solid (14 mg, *quant.*). mp 255 – 261 °C; δ_H (400 MHz, CD_3OD) 4.18 (3H, s), 7.76 (1H, dd, *J* 5.0, 1.5), 8.38 (1H, s), 8.47 (1H, app. s), 8.59 (1H, d, *J* 5.0); *m/z* (MS, ES^-) 203 (100%, $[M\text{-}K]^-$); LCMS tR 9.2 min (254 nm: 97%); accurate mass: found 203.0572, calc. for $C_9H_7N_4O_2^-$ 203.0574.

Methyl 2-(1-ethyl-1*H*-1,2,3-triazol-4-yl)isonicotinate (16c)

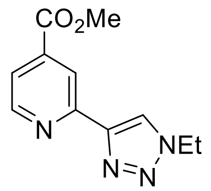

A solution of tetrabutylammonium fluoride in tetrahydrofuran (1M, 0.50 mL, 0.50 mmol) was added to a solution of methyl 2-[(trimethylsilyl)ethynyl]isonicotinate (117 mg, 0.50 mmol), ethyl iodide (44 µL, 86 mg, 0.55 mmol), cupric sulfate pentahydrate (6 mg, 0.03 mmol), sodium azide (39 mg, 0.60 mmol) and (+)-sodium L-ascorbate (11 mg, 0.06 mmol) in 2 mL of a 4:1 mixture of dimethylformamide and water. The resulting solution was heated at 65 °C for 60 h. The reaction mixture was diluted with brine (15 mL) and extracted with ethyl acetate (2 x 12 mL). The organic layers were combined, dried over sodium sulfate and concentrated *in vacuo* to give a brown oil which was purified by flash column chromatography (7:3 ethyl acetate: 30 – 40 petroleum ether) to give **16c** as a brown solid (89 mg, 77%). R_f 0.20 (2:1 30 – 40 petroleum ether:ethyl acetate). mp 106 – 107 °C; δ_H (400 MHz, $CDCl_3$) 1.62 (3H, t, *J* 7.5), 3.97 (3H, s), 4.50 (2H, q, *J* 7.5), 7.77 (1H, dd, *J* 5.0, 1.5), 8.17 (1H, app. s), 8.69 – 8.72 (2H, m); *m/z* (MS, ES^+) 487 (100%, M_2Na^+); LCMS tR 11.5

min (254 nm: 100%); accurate mass: found 255.0850, calc. for $C_{11}H_{12}N_4NaO_2^+$ 255.0852.

Potassium 2-(1-ethyl-1*H*-1,2,3-triazol-4-yl)isonicotinate (14c)

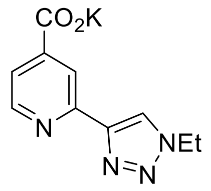

Potassium trimethylsilanolate (14 mg, 0.11 mmol) was added to a solution of methyl 2-(1-ethyl-1*H*-1,2,3-triazol-4-yl)isonicotinate (23 mg, 0.10 mmol) in acetonitrile (2 mL) and the resulting suspension stirred at room temperature for 60 h. The resulting precipitate was filtered, washed with acetonitrile (2 x 3 mL) and dried under vacuum to give **14c** as a beige solid (15 mg, 59%). R_f 0.45 (7:3 dichloromethane:methanol); mp 162 – 164 °C; δ_H (500 MHz, $(CD_3)_2SO$) 1.50 (3H, t, *J* 7.5), 4.45 (2H, q, *J* 7.5), 7.61 (1H, dd, *J* 5.0, 1.5), 8.37 (1H, app. s), 8.50 (1H, dd, *J* 5.0, 0.5), 8.58 (1H, s); *m/z* (MS, ES^-) 217 (100%, $[M-K]^-$); LCMS tR 9.4 min (254 nm: 98%); accurate mass: found 217.0733, calc. for $C_{10}H_9N_4O_2^-$ 217.0731.

Methyl 2-[1-(3-methylphenyl)-1*H*-1,2,3-triazol-4-yl]isonicotinate (16d)

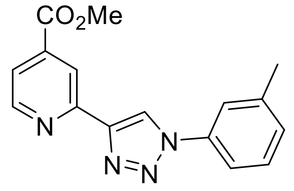

A solution of tetrabutylammonium fluoride in tetrahydrofuran (1M, 0.5 mL, 0.5 mmol) was added to a solution of methyl 2-[(trimethylsilyl)ethynyl]isonicotinate (117 mg, 0.5 mmol), 3-iodotoluene (71 μL, 0.55 mmol), L-proline (12 mg, 0.10 mmol), cupric sulfate pentahydrate (6 mg, 0.03 mmol), sodium azide (39 mg, 0.60 mmol) and (+)-sodium L-ascorbate (11 mg, 0.06 mmol) in 2 mL of a 9:1 mixture of dimethylsulfoxide and water. The resulting solution was heated at 65 °C for 16 h. The reaction mixture was poured into aqueous ammonium hydroxide (1% w/w solution, 0.5 mL) diluted with ethyl acetate (10 mL), washed with brine (3 x 10 mL) and the aqueous layers back-extracted with ethyl acetate (10 mL). The organic layers were combined, dried over sodium sulfate and concentrated *in vacuo* to give a brown oil which was purified by flash column chromatography (9:1 hexane:ethyl acetate) to give **16d** as a brown solid (30 mg, 20%). R_f 0.35 (2:1 hexane:ethyl acetate); mp 143 – 145 °C; δ_H

(400 MHz, $CDCl_3$) 2.48 (3H, s), 4.01 (3H, s), 7.28 (1H, app. d, *J* 8.0) 7.44 (1H, app. t, *J* 8.0), 7.60 (1H, ddd, *J* 8.0, 1.0, 0.5), 7.67 (1H, app. s), 7.82 (1H, dd, *J* 5.0, 1.5), 8.61 (1H, s), 8.76 (1H, dd, *J* 5.0, 1.0), 8.79 (1H, dd, *J* 1.5, 1.0); *m/z* (MS, ES^+) 611 (100%, M_2Na^+); LCMS tR 16.2 min (254 nm: 100%); accurate mass: found 317.1010, calc. for $C_{16}H_{14}N_4NaO_2^+$ 317.1009.

Potassium 2-[1-(3-methylphenyl)-1*H*-1,2,3-triazol-4-yl]isonicotinate (14d)

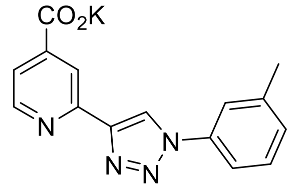

Potassium trimethylsilanolate (15 mg, 0.12 mmol) was added to a solution of 2-[1-(3-methylphenyl)-1*H*-1,2,3-triazol-4-yl]isonicotinate (21 mg, 0.07 mmol) in acetonitrile (4 mL) and the resulting suspension stirred at room temperature for 16 h. The resulting precipitate was filtered, washed with acetonitrile (2 x 3 mL) and dried under vacuum to give **14d** as a yellow solid (20 mg, 88%). R_f 0.15 (9:1 dichloromethane:methanol); mp 198 – 200 °C; δ_H (500 MHz, $(CD_3)_2SO$) 2.43 (3H, s), 7.32 (1H, d, *J* 8.0), 7.49 (1H, t, *J* 8.0), 7.67 (1H, dd, *J* 5.0, 1.5), 7.83 (1H, d, *J* 8.0), 7.90 (1H, s), 8.44 – 8.46 (1H, m), 8.56 (1H, dd, *J* 5.0, 1.0), 9.25 (1H, s); *m/z* (MS, ES^-) 279 (100%, $[M-K]^-$); LCMS tR 13.7 min (254 nm: 100%); accurate mass: found 303.0854, calc. for $C_{15}H_{12}N_4NaO_2^+$ 303.0852.

Methyl 2-(1-benzyl-1*H*-1,2,3-triazol-4-yl)isonicotinate (16e)

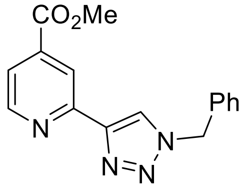

A solution of tetrabutylammonium fluoride in tetrahydrofuran (1M, 1.25 mL, 1.25 mmol) was added to a suspension of methyl 2-[(trimethylsilyl)ethynyl]isonicotinate (291 mg, 1.25 mmol), benzyl azide (166 mg, 1.25 mmol), diisopropylethylamine (44 µL, 0.25 mmol) and cuprous iodide (24 mg, 0.13 mmol) in methanol (2.5 mL) and the resulting suspension stirred at room temperature for 16 h. The reaction mixture was diluted with water (5 mL) then extracted with dichloromethane (3 x 5 mL). The organic layers were combined, evaporated

and purified by flash column chromatography (2:1 heptane:ethyl acetate) to give **16e** as a tan solid (255 mg, 69%). R_f 0.2 (9:1 heptane:ethyl acetate); mp 158 – 160 °C; δ_H (400 MHz, $CDCl_3$) 3.95 (3H, s), 5.57 (2H, s), 7.30 – 7.39 (5H, m), 7.73 (1H, dd, *J* 5.0, 1.5), 8.05 (1H, s), 8.65 (1H, dd, *J* 5.0, 1.0), 8.68 (1H, dd, *J* 1.5, 1.0); *m/z* (MS, ES^+) 295 (100%, MH^+); LCMS tR 14.5 min (254 nm: 98%); accurate mass: found 317.0998, calc. for $C_{16}H_{14}N_4NaO_2^+$ 317.1009.

Potassium 2-(1-benzyl-1*H*-1,2,3-triazol-4-yl)-isonicotinate (14e)

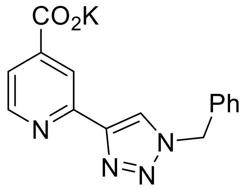

Potassium trimethylsilanolate (24 mg, 0.19 mmol) was added to a solution of methyl 2-(1-benzyl-1*H*-1,2,3-triazol-4-yl)isonicotinate (50 mg, 0.17 mmol) in acetonitrile (2 mL) and the resulting suspension stirred at room temperature for 16 h. The resulting precipitate was filtered, washed with acetonitrile (2 x 2 mL) and dried under vacuum at 30°C for 16 h to give **14e** as an off-white solid (47 mg, 87%). R_f 0.55 (1:4 2M methanolic ammonia:dichloromethane); mp 148 – 151 °C; δ_H (400 MHz, CD_3OD) 5.68 (2H, s), 7.33 – 7.43 (5H, m), 7.76 (1H, dd, *J* 5.0, 1.5), 8.40 (1H, s), 8.49 (1H, dd, *J* 1.5, 0.5), 8.58 (1H, dd, *J* 5.0, 0.5); *m/z* (MS, ES^+) 281 (100%, MH^+); LCMS tR 12.4 min (254 nm: 99%); accurate mass: found 303.0840, calc. for $C_{15}H_{12}N_4NaO_2^+$ 303.0852.

2-[1-(2-Phenylethyl)-1*H*-1,2,3-triazol-4-yl]isonicotinic acid (14f)

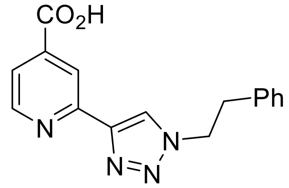

A solution of tetrabutylammonium fluoride (1M in tetrahydrofuran, 0.20 mL, 0.20 mmol) was added to a suspension of methyl 2-[(trimethylsilyl)ethynyl]isonicotinate (47 mg, 0.20 mmol), phenethyl azide (29 mg, 0.20 mmol), diisopropylethylamine (7 μL, 0.04 mmol) and cuprous iodide (4 mg, 0.02 mmol) in methanol (1 mL) and the resulting suspension stirred at room temperature for 16 h. The reaction mixture was diluted with water (5 mL) then

extracted with dichloromethane (3 x 5 mL). The organic layers were combined, evaporated and concentrated *in vacuo*. Potassium trimethylsilanolate (28 mg, 0.22 mmol) was added as a solution in acetonitrile (2 mL) and the resulting suspension stirred at room temperature for 2 h. The precipitate was filtered and washed with acetonitrile (2 x 2 mL) then dried under vacuum at 30°C for 16 h and purified by reverse phase HPLC (0.1% formic acid in water → 0.1% formic acid in acetonitrile) to give **14f** as a beige solid (21 mg, 32%). *m/z* (MS, ES^+) 295 (100%, MH^+); LCMS (system B) tR 2.9 min (225 nm: 84%; ELSD: 98%).

2-[1-(2-Phenoxyethyl)-1*H*-1,2,3-triazol-4-yl]isonicotinic acid (14g)

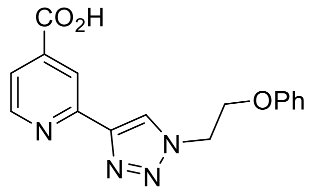

A solution of tetrabutylammonium fluoride (1M in tetrahydrofuran, 0.20 mL, 0.20 mmol) was added to a suspension of methyl 2-[(trimethylsilyl)ethynyl]isonicotinate (47 mg, 0.20 mmol), 2-phenoxyethyl azide (33 mg, 0.20 mmol), diisopropylethylamine (7 µL, 0.04 mmol) and cuprous iodide (4 mg, 0.02 mmol) in methanol (1 mL) and the resulting suspension stirred at room temperature for 16 h. The reaction mixture was diluted with water (5 mL) then extracted with dichloromethane (3 x 5 mL). The organic layers were combined, evaporated and concentrated *in vacuo*. Potassium trimethylsilanolate (28 mg, 0.22 mmol) was added as a solution in acetonitrile (2 mL) and the resulting suspension stirred at room temperature for 2 h. The precipitate was filtered and washed with acetonitrile (2 x 2 mL) then dried under vacuum at 30°C for 16 h and purified by reverse phase HPLC (0.1% formic acid in water → 0.1% formic acid in acetonitrile) to give **14g** as a beige solid (35 mg, 50%). *m/z* (MS, ES^+) 311 (100%, MH^+); LCMS (system B) tR 2.9 min (225 nm: 96%; ELSD: 100%).

2-{1-[1-(Ethoxycarbonyl)piperidin-4-yl]-1*H*-1,2,3-triazol-4-yl}isonicotinic acid (14h)

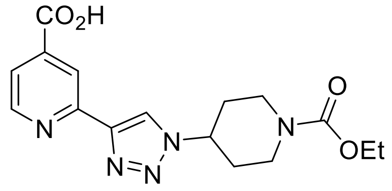

A solution of tetrabutylammonium fluoride (1M in tetrahydrofuran, 0.20 mL, 0.20 mmol)

was added to a suspension of methyl 2-[(trimethylsilyl)ethynyl]isonicotinate (47 mg, 0.20 mmol), ethyl 4-azidopiperidine-1-carboxylate (40 mg, 0.20 mmol), diisopropylethylamine (7 μL, 0.04 mmol) and cuprous iodide (4 mg, 0.02 mmol) in methanol (1 mL) and the resulting suspension stirred at room temperature for 16 h. The reaction mixture was diluted with water (5 mL) then extracted with dichloromethane (3 x 5 mL). The organic layers were combined, evaporated and concentrated *in vacuo*. Potassium trimethylsilanolate (28 mg, 0.22 mmol) was added as a solution in acetonitrile (2 mL) and the resulting suspension stirred at room temperature for 2 h. The precipitate was filtered and washed with acetonitrile (2 x 2 mL) then dried under vacuum at 30°C for 16 h and purified by reverse phase HPLC (0.1% formic acid in water → 0.1% formic acid in acetonitrile) to give **14h** as a brown solid (11 mg, 14%). *m/z* (MS, ES^+) 346 (100%, MH^+); LCMS (system B) tR 2.9 min (225 nm: 99%; ELSD: 100%).

Representative Syntheses of Amide Derivatives

Compound **31** was synthesized according to a literature procedure and its physical and spectroscopic properties are in agreement to those reported.[3]

***tert*-Butyl (3*S*)-3-[(methylsulfonyl)oxy]piperidine-1-carboxylate (31)**

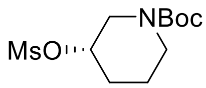

Methanesulfonyl chloride (5.0 mL, 64.6 mmol) was added dropwise over 5 min to a solution of *tert*-butyl (3*S*)-3-hydroxypiperidine-1-carboxylate (10.79 g, 53.6 mmol) and triethylamine (15.0 mL, 108 mmol) in ethyl acetate (100 mL) at -5 °C and the resulting suspension stirred at -5 °C for 1 h. Water was added (100 mL), the mixture allowed to warm to room temperature, the layers separated and the organic layer washed with hydrochloric acid (55 mL of a 1M aq solution) followed by sodium hydrogencarbonate (100 mL of a saturated solution). The organic layer was dried over sodium sulfate and concentrated *in vacuo* to give **31** as a white solid (14.44 g, 96%). mp 92 – 97 °C; δ_H (400 MHz, $CDCl_3$) 1.43 (9H, s), 1.46 – 1.82 (2H, m), 1.82 – 1.99 (2H, m), 3.03 (3H, s), 3.24 – 3.48 (2H, m), 3.53 – 3.67 (2H, m), 4.64 – 4.74 (1H, m); *m/z* (MS, EI^+) 279 (100%, $[M]^+$); elemental analysis: $C_{11}H_{21}NO_5S$ requires C, 47.3; H, 7.6; N, 5.0%; found C, 47.5; H, 7.7; N, 5.1%; $[\alpha]_D^{20}$ −0.4 (*c* 1.0 in $CHCl_3$).

Methyl 2-{1-[(3*R*)-1-(*tert*-butoxycarbonyl)piperidin-3-yl]-1*H*-1,2,3-triazol-4-yl}isonicotinate (33)

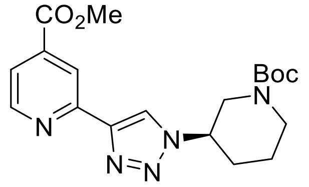

Sodium azide (1.54 g, 23.7 mmol) was added to a solution of *tert*-butyl (3*S*)-3-[(methylsulfonyl)oxy]piperidine-1-carboxylate (3.35 g, 12.0 mmol) in dimethylformamide (25 mL) and the resulting suspension heated at 75 °C for 6 h. The reaction mixture was cooled to room temperature then diisopropylethylamine (0.33 mL, 1.89 mmol), cuprous iodide (180 mg, 0.96 mmol) and a solution of tetrabutylammonium fluoride in tetrahydrofuran (1M, 12.0 mL, 12.0 mmol) were added. A solution of methyl 2-[(trimethylsilyl)ethynyl]isonicotinate (2.32 g, 9.94 mmol) in dimethylformamide (10 mL) was added dropwise over 20 minutes. The reaction mixture was stirred at room temperature for 16 h then diluted with brine (100 mL) and extracted with ethyl acetate (3 x 100 mL). The organic layers were washed with brine (2 x 100 mL) then combined, dried over sodium sulfate, concentrated *in vacuo* and purified by flash column chromatography (cyclohexane → 55% ethyl acetate in cyclohexane) to give **33** as an orange foam (1.84 g, 48%). R_f 0.50 (3:1 ethyl acetate:cyclohexane); δ_H (400 MHz, CD_3OD) 1.44 (9H, s), 1.61 – 1.75 (1H, m), 1.83 – 1.95 (1H, m), 2.19 – 2.23 (2H, m), 3.13 – 3.28 (1H, m), 3.41 – 3.94 (2H, m), 3.98 (3H, s), 4.09 – 4.34 (1H, m), 4.62 – 4.75 (1H, m), 7.80 (1H, dd, *J* 5.0, 1.5), 8.52 (1H, s), 8.58 (1H, s), 8.72 (1H, d, *J* 5.0); *m/z* (MS, ES^+) 388 (100%, MH^+); LCMS tR 15.4 min (254 nm: 98%); accurate mass: found 410.1785, calc. for $C_{19}H_{25}N_5NaO_4^+$ 410.1799.

Methyl 2-{1-[(3*R*)-piperidin-3-yl]-1*H*-1,2,3-triazol-4-yl}isonicotinate (s3)

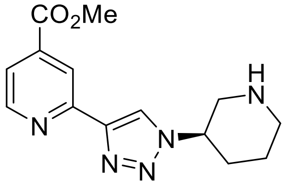

Trifluoroacetic acid (2.0 mL, 25.6 mmol) was added to a solution of methyl 2-{1-[(3*R*)-1-(*tert*-butoxycarbonyl)piperidin-3-yl]-1*H*-1,2,3-triazol-4-yl}isonicotinate (1.10 g, 2.84 mmol)

in dichloromethane (4 mL) and the resulting solution stirred at room temperature for 16 h. The reaction mixture was concentrated *in vacuo* then purified on a 10 g strong cation exchange column, washing with methanol (100 mL) then eluting with methanolic ammonia (100 mL of a 2M solution) to give **s3** as an orange gum (789 mg, 97%). R_f 0.45 (9:1 dichloromethane:2M methanolic ammonia); δ_H (400 MHz, CD_3OD) 1.65 – 1.78 (1H, m), 1.86 – 1.94 (1H, m), 2.06 – 2.18 (1H, m), 2.29 – 2.39 (1H, m), 2.64 – 2.75 (1H, m), 3.00 – 3.08 (2H, m), 3.37 – 3.44 (1H, m), 3.98 (3H, s), 4.61 – 4.70 (1H, m), 7.81 (1H, d, *J* 5.0,), 8.51 (1H, s), 8.56 (1H, s), 8.72 (1H, d, *J* 5.0); *m/z* (MS, ES^+) 288 (100%, MH^+); LCMS (system E) tR 2.8 min (254 nm: 97%); accurate mass: found 310.1263, calc. for $C_{14}H_{17}N_5NaO_2^+$ 310.1274.

Methyl 2-{1-[(3*R*)-1-benzoylpiperidin-3-yl]-1*H*-1,2,3-triazol-4-yl}isonicotinate (s4)

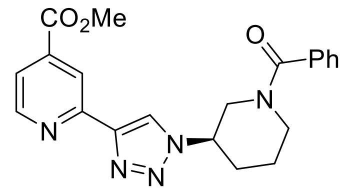

Benzoyl chloride (242 µL, 2.09 mmol) was added to a solution of methyl 2-{1-[(3*R*)-piperidin-3-yl]-1*H*-1,2,3-triazol-4-yl}isonicotinate (599 mg, 2.09 mmol) and triethylamine (0.30 mL, 2.09 mmol) in acetonitrile (15 mL) and the resulting solution stirred at room temperature for 1.5 h. The reaction mixture was diluted with water (20 mL), basified to pH 10 with sodium hydrogencarbonate (aqueous, saturated) and extracted with dichloromethane (2 x 20 mL). The organic layers were combined, dried over sodium sulfate then concentrated *in vacuo* to give **s4** as a beige foam (856 mg, *quant.*). δ_H (200 MHz, $(CD_3)_2SO$) 1.49 – 2.00 (2H, m), 2.08 – 2.40 (2H, m), 3.03 – 3.85 (3H, m), 3.94 (3H, s), 4.00 – 4.69 (1H, m), 4.71 – 4.93 (1H, m), 7.35 – 7.51 (5H, m, *Ph*), 7.78 (1H, dd *J* 5.0, 1.5), 8.44 (1H, app. s), 8.74 – 8.91 (2H, m); *m/z* (MS, ES^+) 392 (100%, MH^+); LCMS tR (system E) 6.8 min (254 nm: 96%); accurate mass: found 414.1525, calc. for $C_{21}H_{21}N_5NaO_3^+$ 414.1537.

2-{1-[(3*R*)-1-Benzoylpiperidin-3-yl]-1*H*-1,2,3-triazol-4-yl}isonicotinic acid hemitrifluoroacetate (35)

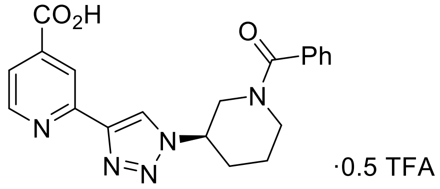

Lithium hydroxide (2.0 mL of a 1M aq solution, 2.0 mmol) was added to a solution of methyl 2-{1-[(3*R*)-1-benzoylpiperidin-3-yl]-1*H*-1,2,3-triazol-4-yl}isonicotinate in acetonitrile (10 mL) and the resulting solution stirred at room temperature for 3 h. The reaction mixture was concentrated *in vacuo* and taken up in 10 mL of an 4:1 mixture of water:acetonitrile. 0.4 mL of trifluoroacetic acid was added and the resulting precipitate filtered, washing with water (2 x 10 mL). The precipitate was dried *in vacuo* to give **35** as a white solid (664 mg, 85%). mp 264 – 250 °C; δ_H (500 MHz, $(CD_3)_2SO$) 1.58 – 2.02 (2H, m), 2.17 – 2.35 (2H, m), 3.07 – 4.67 (4H, m), 4.77 – 4.87 (1H, m), 7.35 – 7.53 (5H, m), 7.77 (1H, app. s), 8.44 (1H, s), 8.66 – 8.92 (2H, m); *m/z* (MS, ES^-) 376 (100%, $[M-H]^-$); LCMS tR 11.7 min (254 nm: 99%); accurate mass: found 400.1377, calc. for $C_{20}H_{19}N_5O_3Na^+$ 400.1380; elemental analysis: $C_{20}H_{19}N_5O_3$.0.5 $C_2HF_3O_2$ requires C, 58.1; H, 4.5; N, 16.1%; found C, 57.9; H, 4.7; N, 15.8%.

2-[1-(1-Propionylpiperidin-4-yl)-1*H*-1,2,3-triazol-4-yl]isonicotinic acid (21a)

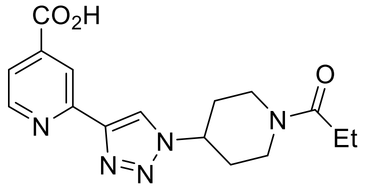

Synthesised as described above for **35** to give **21a** as a white solid. LCMS (system D): tR 0.89 min (280nm: 100%); *m/z* 330 (MH^+).

2-[1-(1-Butyrylpiperidin-4-yl)-1*H*-1,2,3-triazol-4-yl]isonicotinic acid (21b)

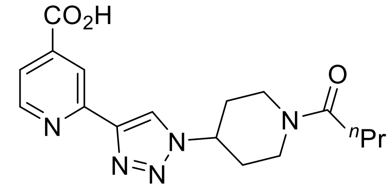

Synthesised as described above for **35** to give **21b** as a white solid. LCMS (system D): tR 0.95 min (280nm: 100%); *m/z* 344 (MH^+).

2-[1-(1-Benzoylpiperidin-4-yl)-1*H*-1,2,3-triazol-4-yl]isonicotinic acid (21c)

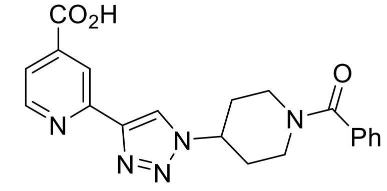

Synthesised as described above for **35** to give **21c** as a white solid. LCMS (system C): tR 1.42 min (280nm: 100%); *m/z* 378 (MH^+).

2-[1-(1-Phenylacetylpiperidin-4-yl)-1*H*-1,2,3-triazol-4-yl]isonicotinic acid (21d)

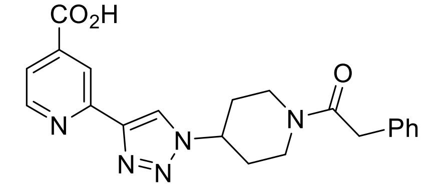

Synthesised as described above for **35** to give **21d** as a white solid. LCMS (system C): tR 1.47 min (280nm: 100%); *m/z* 392 (MH^+).

2-[1-(1-Phenylpropanoylpiperidin-4-yl)-1*H*-1,2,3-triazol-4-yl]isonicotinic acid (21e)

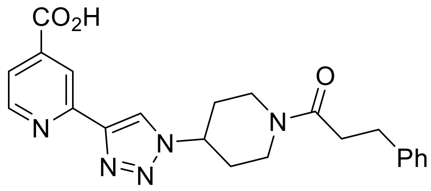

Synthesised as described above for **35** to give **21e** as a white solid. LCMS (system C): tR

1.56 min (280nm: 100%); *m/z* 406 (MH^+).

2-[1-(1-Acetylazetidin-3-yl)-1*H*-1,2,3-triazol-4-yl]isonicotinic acid (28a)

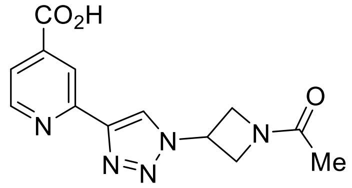

Synthesised as described above for **35** to give **28a** as a white solid. LCMS (system D): tR 1.00 min (280nm: 100%); *m/z* 288 (MH^+).

2-[1-(1-Propionylazetidin-3-yl)-1*H*-1,2,3-triazol-4-yl]isonicotinic acid (28b)

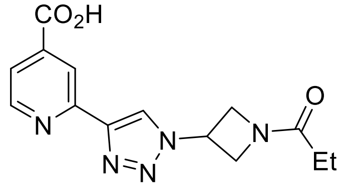

Synthesised as described above for **35** to give **28b** as a white solid. LCMS (system D): tR 1.11 min (280nm: 100%); *m/z* 302 (MH^+).

2-[1-(1-Butyrylazetidin-3-yl)-1*H*-1,2,3-triazol-4-yl]isonicotinic acid (28c)

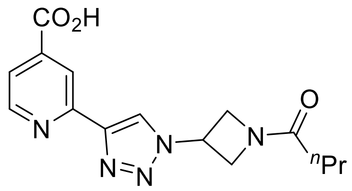

Synthesised as described above for **35** to give **28c** as a white solid. LCMS (system D): tR 1.23 min (280nm: 98.5%); *m/z* 316 (MH^+).

2-[1-(1-Benzoylazetidin-3-yl)-1*H*-1,2,3-triazol-4-yl]isonicotinic acid (28d)

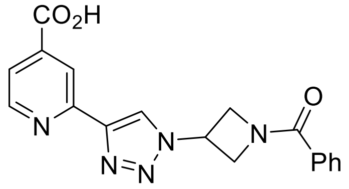

Synthesised as described above for **35** to give **28d** as a white solid. LCMS (system D): tR 1.35 min (280nm: 100%); *m/z* 350 (MH^+).

2-[1-(1-Phenylacetylazetidin-3-yl)-1*H*-1,2,3-triazol-4-yl]isonicotinic acid (28e)

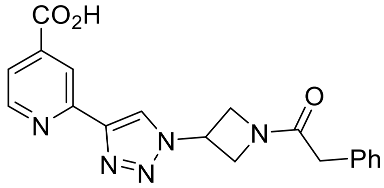

Synthesised as described above for **35** to give **28e** as a white solid. LCMS (system D): tR 1.42 min (280nm: 100%); *m/z* 364 (MH^+).

2-[1-(1-Phenylpropanoylazetidin-3-yl)-1*H*-1,2,3-triazol-4-yl]isonicotinic acid (28f)

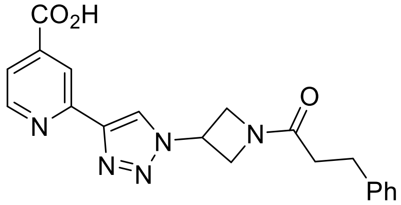

Synthesised as described above for **35** to give **28f** as a white solid. LCMS (system D): tR 1.48 min (280nm: 100%); *m/z* 378 (MH^+).

***rac*-2-[1-(1-Butyrylpyrrolidin-3-yl)-1*H*-1,2,3-triazol-4-yl]isonicotinic acid (28g)**

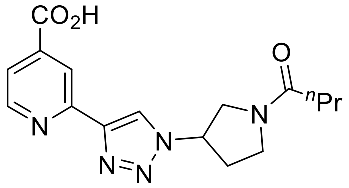

Synthesised as described above for **35** to give **28g** as a white solid. LCMS (system D): tR

1.38 min (280nm: 100%); *m/z* 330 (MH^+).

rac-2-[1-(1-Benzoylpyrrolidin-3-yl)-1_H_-1,2,3-triazol-4-yl]isonicotinic acid (28h)

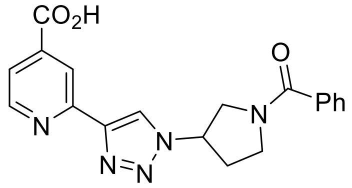

Synthesised as described above for **35** to give **28h** as a white solid. LCMS (system C): tR 1.37 min (280nm: 96.0%); *m/z* 364 (MH^+).

rac-2-[1-(1-Phenylacetylpyrrolidin-3-yl)-1_H_-1,2,3-triazol-4-yl]isonicotinic acid (28i)

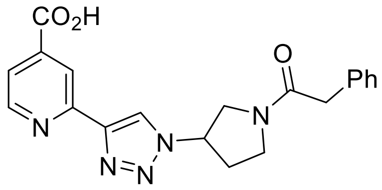

Synthesised as described above for **35** to give **28i** as a white solid. LCMS (Method C): tR 1.40 min (280nm: 98.5%); *m/z* (ES^-) 376 (100%, $[M-H]^-$).

rac-2-[1-(1-Phenylpropanoylpyrrolidin-3-yl)-1_H_-1,2,3-triazol-4-yl]isonicotinic acid (28j)

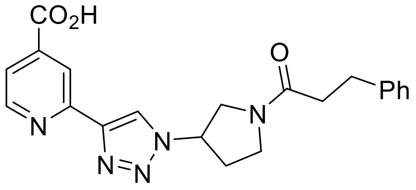

Synthesised as described above for **35** to give **28j** as a white solid. LCMS (system C): tR 1.50 min (280nm: 100%); *m/z* 392 (MH^+).

rac-2-[1-(1-Acetylpiperidin-3-yl)-1_H_-1,2,3-triazol-4-yl]isonicotinic acid (28k)

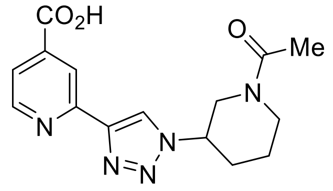

Synthesised as described above for **35** to give **28k** as a yellow solid. LCMS (system C): tR 1.15 min (280nm: 100%); *m/z* 316 (MH^+).

rac-2-[1-(1-Propionylpiperidin-3-yl)-1_H_-1,2,3-triazol-4-yl]isonicotinic acid (28l)

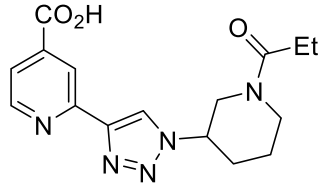

Synthesised as described above for **35** to give **28l** as a yellow solid. LCMS (system C): tR 1.28 min (280nm: 100%); *m/z* 330 (MH^+).

rac-2-[1-(1-Butyrylpiperidin-3-yl)-1_H_-1,2,3-triazol-4-yl]isonicotinic acid (28m)

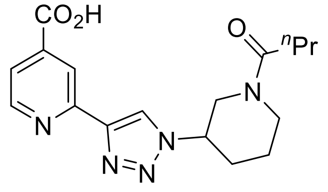

Synthesised as described above for **35** to give **28m** as a yellow solid. LCMS (system C): tR 1.39 min (280nm: 100%); *m/z* 344 (MH^+).

rac-2-[1-(1-Benzoylpiperidin-3-yl)-1_H_-1,2,3-triazol-4-yl]isonicotinic acid (28n)

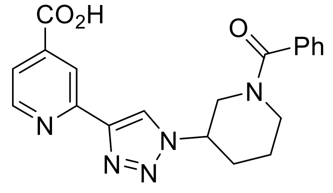

Synthesised as described above for **35** to give **28n** as a white solid. LCMS (system C): tR

1.45 min (280nm: 100%); *m/z* 378 (MH^+).

***rac*-2-[1-(1-Phenylacetylpiperidin-3-yl)-1*H*-1,2,3-triazol-4-yl]isonicotinic acid (28o)**

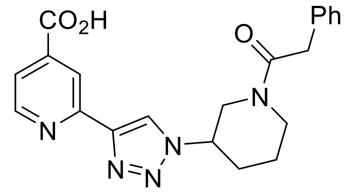

Synthesised as described above for **35** to give **28o** as a yellow solid. LCMS (system C): tR 1.50 min (280nm: 100%); *m/z* 392 (MH^+).

***rac*-2-[1-(1-Phenylpropanoylpiperidin-3-yl)-1*H*-1,2,3-triazol-4-yl]isonicotinic acid (28p)**

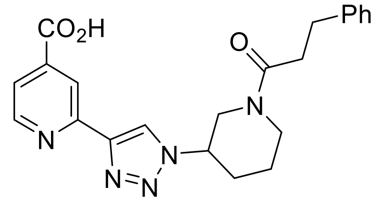

Synthesised as described above for **35** to give **28p** as a white solid. LCMS (system C): tR 1.61 min (280nm: 100%); *m/z* 406 (MH^+).

Compound **32** was synthesized according to a literature procedure and its physical and spectroscopic properties are in agreement to those reported.[3]

***tert*-Butyl (3*R*)-3-[(methylsulfonyl)oxy]piperidine-1-carboxylate (32)**

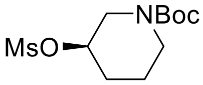

Methanesulfonyl chloride (0.19 mL, 2.40 mmol) was added dropwise over 5 min to a solution of *tert*-butyl (3*R*)-3-hydroxypiperidine-1-carboxylate (403 mg, 2.00 mmol) and triethylamine (0.56 mL, 4.00 mmol) in ethyl acetate (5.5 mL) at -5 °C and the resulting suspension stirred at -5 °C for 1 h. Water was added (6 mL), the layers separated and the organic layer washed with hydrochloric acid (2 mL of a 1M aq solution) followed by sodium hydrogencarbonate (6 mL of a saturated solution). The organic layer was dried over sodium sulfate and concentrated *in vacuo* to give **32** as a white solid (546 mg, 98%). mp 91 – 96 °C; δ_H (400

MHz, $CDCl_3$) 1.45 (9H, s), 1.48 – 1.85 (2H, m), 1.86 – 2.00 (2H, m), 3.04 (3H, s), 3.24 – 3.47 (2H, m), 3.55 – 3.67 (2H, m), 4.66 – 4.74 (1H, m); *m/z* (MS, EI^+) 279 (100%, $[M]^+$); elemental analysis: $C_{11}H_{21}NO_5S$ requires C, 47.3; H, 7.6; N, 5.0%; found C, 47.15; H, 7.7; N, 5.1%; $[\alpha]_D^{20}$ +0.4 (*c* 1.0 in $CHCl_3$).

Methyl 2-{1-[(3*S*)-1-(*tert*-butoxycarbonyl)piperidin-3-yl]-1*H*-1,2,3-triazol-4-yl}isonicotinate (34)

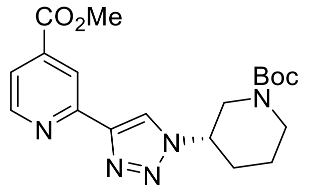

Sodium azide (186 mg, 2.86 mmol) was added to a solution of *tert*-butyl (3*R*)-3-[(methylsulfonyl)oxy]piperidine-1-carboxylate (400 mg, 1.43 mmol) in dimethylformamide (5 mL) and the resulting suspension heated at 75 °C for 5 h. The reaction mixture was cooled to room temperature then diisopropylethylamine (45 µL, 0.26 mmol), cuprous iodide (25 mg, 0.13 mmol) and methyl 2-ethynylisonicotinate (210 mg, 1.30 mmol) were added. The reaction mixture was stirred at room temperature for 16 h then diluted with brine (20 mL) and extracted with ethyl acetate (2 x 20 mL). The organic layers were washed with brine (20 mL) then combined, evaporated and purified by flash column chromatography (dichloromethane → 4:1 dichloromethane:ethyl acetate) to give **34** as a white foam (202 mg, contaminated with 16% dimethylformamide w/w, 34%). R_f 0.5 (3:2 dichloromethane:ethyl acetate; δ_H (400 MHz, $CDCl_3$) 1.42 (9H, s), 1.56 – 1.70 (1H, m), 1.76 – 1.87 (1H, m), 2.08 – 2.20 (1H, m), 2.24 – 2.34 (1H, m), 2.97 – 3.07 (1H, m), 3.30 – 3.46 (1H, m), 3.85 – 3.92 (1H, m), 3.93 (3H, s), 4.18 – 4.37 (1H, m), 4.52 – 4.61 (1H, m), 7.74 (1H, d, *J* 4.0), 8.26 (1H, s), 8.64 – 8.68 (2H, m); *m/z* (MS, ES^+) 410 (100%, MNa^+); LCMS tR 15.5 min (254 nm: 96%); accurate mass: found 410.1789, calc. for $C_{19}H_{25}N_5NaO_4^+$ 410.1799.

2-{1-[(3*S*)-1-Benzoylpiperidin-3-yl]-1*H*-1,2,3-triazol-4-yl}isonicotinic acid hemitrifluoroacetate (36)

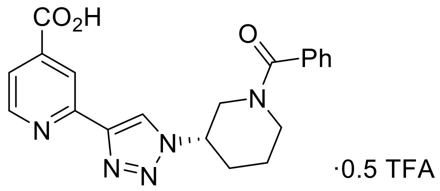

Trifluoroacetic acid (120 μL, 0.20 mmol) was added to a solution of methyl 2-{1-[(3*S*)-1-(*tert*-butoxycarbonyl)piperidin-3-yl]-1*H*-1,2,3-triazol-4-yl}isonicotinate (100 mg, 84% w/w, 0.17 mmol) in dichloromethane (0.5 mL) and the resulting solution stirred at room temperature for 16 h. The reaction mixture was concentrated *in vacuo* then dissolved in acetonitrile (2 mL). Benzoyl chloride (30 μL, 0.26 mmol) and triethylamine (0.22 mL, 0.73 mmol) were added and the resulting solution stirred at room temperature for 2.5 h. Lithium hydroxide (2.0 mL of a 1M aq solution, 2.0 mmol) was added and the resulting solution stirred at room temperature for 2 h. The reaction mixture was acidified to pH 1 with trifluoroacetic acid then concentrated *in vacuo* and purified by reverse phase chromatography (C18 column, eluting with 0.1% trifluoroacetic acid in water → 0.1% trifluoroacetic acid in 3:2 acetonitrile:water) to give **36** as an off-white solid (70 mg, 81%). mp 254 – 263 °C; δ_H (500 MHz, $(CD_3)_2SO$) 1.57 – 2.00 (2H, m), 2.16 – 2.35 (2H, m), 3.06 – 4.68 (4H, m), 4.77 – 4.85 (1H, m), 7.35 – 7.52 (5H, m), 7.77 (1H, d *J* 4.0), 8.44 (1H, s), 8.68 – 8.90 (2H, m); *m/z* (MS, ES^+) 400 (100%, MNa^+); LCMS (system E) tR 3.9 min (254 nm: 95%); accurate mass: found 400.1372, calc. for $C_{20}H_{19}N_5NaO_3^+$ 400.1380.

Methyl 2-{1-[(3*R*)-1-benzoylpiperidin-3-yl]-1*H*-1,2,3-triazol-4-yl}isonicotinate (37)

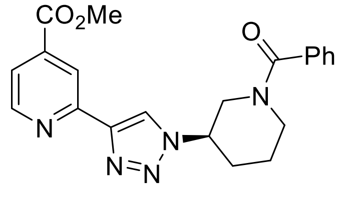

Methyl iodide (13.8 μL, 0.22 mmol) was added to a suspension of 2-{1-[(3*R*)-1-benzoylpiperidin-3-yl]-1*H*-1,2,3-triazol-4-yl}isonicotinic acid hemitrifluoroacetate (87 mg, 0.20 mmol) and sodium hydrogencarbonate (50.7 mg, 0.60 mmol) in dimethylformamide (0.9 mL) and the resulting suspension stirred at room temperature for 16 h. Further methyl iodide (13.8 μL, 0.22 mmol) was added and the resulting suspension stirred at room temperature for

16 h. Further methyl iodide (13.8 µL, 0.22 mmol) was added and the resulting suspension stirred at room temperature for 6 h. Ethyl acetate (5 mL) was added and the resulting suspension washed with brine (5 x 5 mL). The aqueous layers were back-extracted with ethyl acetate (5 mL). The organics layers were combined, dried over sodium sulfate, concentrated *in vacuo* and purified by flash column chromatography (dichloromethane → 6% methanol in dichloromethane) to give **37** as an off-white gum (80 mg, *quant.*). δ_H (400 MHz, $(CD_3)_2SO$) 1.54 – 1.98 (2H, m), 2.14 – 2.36 (2H, m), 3.05 – 3.75 (3H, m), 3.92 (3H, s), 4.01 – 4.70 (1H, m), 4.73 – 4.88 (1H, m), 7.34 – 7.52 (5H, m), 7.75 (1H, dd *J* 5.0, 1.5), 8.43 (1H, app. s), 8.68 – 8.89 (2H, m); *m/z* (MS, ES^+) 392 (100%, MH^+); LCMS tR (system E) 6.8 min (254 nm: 99%); *ee* 98% (tR (minor) 26.3 min [254 nm: 1%]; tR (major) 53.7 min [254 nm: 99%]).

Methyl 2-{1-[(3*S*)-1-benzoylpiperidin-3-yl]-1*H*-1,2,3-triazol-4-yl}isonicotinate (38)

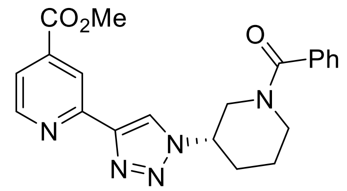

Methyl iodide (4.6 µL, 0.073 mmol) was added to a suspension of 2-{1-[(3*S*)-1-benzoylpiperidin-3-yl]-1*H*-1,2,3-triazol-4-yl}isonicotinic acid hemitrifluoroacetate (29 mg, 0.067 mmol) and sodium hydrogencarbonate (16.9 mg, 0.20 mmol) in dimethylformamide (0.5 mL) and the resulting suspension stirred at room temperature for 16 h. Further methyl iodide (4.6 µL, 0.073 mmol) was added and the resulting suspension stirred at room temperature for 16 h. Further methyl iodide (4.6 µL, 0.073 mmol) was added and the resulting suspension stirred at room temperature for 6 h. Ethyl acetate (5 mL) was added and the resulting suspension washed with brine (5 x 5 mL). The aqueous layers were back-extracted with ethyl acetate (5 mL). The organics layers were combined, dried over sodium sulfate, concentrated *in vacuo* and purified by flash column chromatography (dichloromethane → 6% methanol in dichloromethane) to **38** as a white foam (17 mg, 65%). δ_H (400 MHz, $(CD_3)_2SO$) 1.56 – 2.01 (2H, m), 2.16 – 2.37 (2H, m), 3.02 – 3.90 (3H, m), 3.94 (3H, s), 3.97 – 4.69 (1H, m), 4.73 – 4.90 (1H, m), 7.32 – 7.55 (5H, m), 7.79 (1H, d *J* 4.0), 8.45 (1H, s), 8.67 – 8.95 (2H, m); *m/z* (MS, ES^+) 392 (100%, MH^+); LCMS tR (system E) 6.8 min (254 nm: 100%); *ee* 98% (tR (major) 25.8 min [254 nm: 99%]; tR (minor) 44.9 min [254 nm: 1%]).

2. Lysine Demethylase (KDM) Catalytic Turnover Assay using Alphascreen

All reagents were from Sigma Aldrich unless otherwise stated and were of the highest purity. Bovine serum albumin (BSA) used in the alphascreen assays was essentially free from fatty acids and globulin (Sigma A7030). Hydroxy Ethyl Piperazine Ethane Sulfonic acid (HEPES) buffer was from Life Technologies. Anti Histone-H3-K9Me1 (ab8896), Anti Histone-H3-K9Me2 (ab1220) and Anti-Histone-H3-K36Me1 (ab9048) were from Abcam, Anti Histone-H3-K27Me2 (07-452) antibody was from Millipore and Anti-Histone-H3-K4Me2 (9726S) antibody was from Cell Signaling Technology. Alphascreen General IgG detection kit was from Perkin Elmer. The peptide substrates H3(1-21)K9Me3-GGK-Biotin (64360, JMJD2 substrate), H3(1-21)K4Me3-GGK-Biotin (64192, JARID substrate), H3(1-21)K9Me2-GGK-Biotin (64359, JMJD1A substrate) and H3(21-44)K27Me3-GK-Biotin (64367, JMJD3 substrate) were from Anaspec. For the FBXL11 assay the peptide substrates Biotin-H3(28-48)K36Me2 and H3(28-48)K36Me2 were synthesized in house. Stocks of enzyme cofactors were prepared fresh each day. Ferrous Ammonium Sulphate (FAS) was made up fresh every day by dissolving 50 – 100 mg in 20 mM HCl to a concentration of 400 mM, this was then diluted to 1 mM in deionized water.

KDM enzyme assays were performed as previously described (Kawamura et al., 2009; Rose et al., 2012). Essentially enzyme assays were carried out in 384-well white proxiplates in 10 μl of assay buffer (50 mM HEPES pH 7.5, 0.1% (w/v) BSA and 0.01 % (v/v) Tween-20). For IC_{50} potency determinations titrations of compound were transferred to dry 384-well proxiplates using an Echo-550 Acoustic Dispenser (Labcyte Technologies). Enzyme (5 μl) was dispensed into each well using a Multidrop (Thermo Scientific) and allowed to incubate with the compounds for 15 min at room temperature. After transfer of substrate (5 μL) the enzyme reaction was progressed for the indicated time period (see **Table S1** for final

concentrations and assay parameters). The enzyme reaction was stopped after the indicated time by addition of 5 μL of Stop Solution (30 mM EDTA, 800 mM NaCl in assay buffer). Streptavidin Donor beads (0.08 mg/ml) and Protein-A conjugated acceptor beads (0.08 mg/ml) were pre-incubated for 1 hour with an antibody to the product methyl mark (**Table S1**) and the presence of biotin-H3-product was detected by addition of 5 μL of the pre-incubated alphascreen beads (final concentrations of 0.02 mg/ml with respect to acceptor and donor beads). Detection was allowed to proceed for 1 hour at room temperature and the assay plates read in a BMG Labtech Pherastar FS plate reader. Data were normalized to the no enzyme control and the IC_{50} determined from the nonlinear regression curve fit using GraphPad Prism 5.

Table S1: Demethylase alphascreen assays parameters and component concentrations.

	**JMJD2C**	**JMJD2E**	**JMJD3**	**JMJD1A**	**FBXL11**	**JARID1C**
Enzyme (nM)	1	1	1	0.2	25	0.5
2-OG (μM)	10	10	10	5	10	5
L-AA (μM)	100	100	100	100	100	100
FAS (μM)	1	1	10	10	10	10
Peptide (μM)	0.03	0.03	0.06	0.06	0.1*	0.1
Substrate Incubation Time	15 min	10 min	5 min	5 min	30 min	20 min
Antibody	0.05 μg/ml	0.05 μg/ml	0.4 μg/ml	0.03 μg/ml	0.005 μg/ml	1:4000

*Blend of Biotinylated H3K36Me2 (0.02 μM) and Non-Biotinylated (0.08 μM)

3. Crystallography

Crystals of JMJD2A were grown as described.[4] The crystals were soaked with compound 14a by mixing 0.5 μL of 10mM compound **14a** (in ethylene glycol) with 1.5 μL reservoir solution (0.1M BIS-TRIS pH 5.9, 0.15M ammonium sulfate, 11% PEG3350) and adding it to the

crystals. The crystals were directly flash frozen in liquid nitrogen after incubating them for 12 hours. A dataset was collected at the Diamond Light Source, beamline I04-1 with a Pilatus 2M CCD detector at 0.92Å. Data were integrated with XDS[5] and scaled with AIMLESS.[6] Ligand dictionaries for compound **14a** where generated with GRADE.[7] Refinement was done with PHENIX[8] and after several cycles of manual rebuilding with COOT,[9] the model converged to a Rfactor/ Rfree of 20.7 and 25.9%, respectively. The quality of the model was validated with MOLPROBITY.[10] For data collection and refinement statistics see **Table S2**.

Table S2. Data collection and refinement statistics.

	**JMJD2A-14a**
PDB Acquisition Code	4URA
**Data collection**	
Diffraction source	Beamline I04-1, Diamond Light Source
Space group	$P2_12_12$
Cell dimensions	
a,b,c (Å)	101.04,149.66,57.86
α,β,γ (°)	90,90,90
Resolution (Å) (outer shell)	60 – 2.23 (2.29 – 2.23)
No. of molecules/ASU	2
No. of unique reflections	43239 (3082)
Completeness (%)	99.2 (97.6)
Redundancy	6.7 (6.3)
R_{sym}	0.087 (0.827)
Mean I/σ(I)	15.9 (2.3)
**Refinement**	
R_{factor}	0.207
R_{free}	0.259
R.m.s.d.	
Bond length (Å)	0.008
Bond angle (°)	1.13
No. of atoms	
Protein	2780 (A); 2756 (B)
compound **14a**	14 (A); 14 (B)
Ligand/ion	18
Water	185
$\langle B_{factor} \rangle$ (Å^2)	
Protein	42.6 (A); 45.2 (B)
compound **14a**	37.5 (A); 37.6 (B)
Ligand/ion	51.8
Water	40.7

Numbers in parentheses refer to the highest resolution shell.

R_{sym} is the unweighted R-value on I between symmetry mates.

$R_{factor} = \Sigma hkl \mid |F_{obs} (hkl)| - k |F_{calc} (hkl)| \mid / \Sigma hkl |F_{obs} (hkl)|$ for the working set of reflections; R_{free} is the R-value for 5% of the reflections excluded from refinement.

References

1. *WO Pat.,* S. W. Bagley, D. A. Griffith, D. W.-S. Kung, WO 2011/058473 A1.
2. J. C. Loren, A. Krasiński, V. V. Fokin and K. B. Sharpless, *Synlett*, 2005, 2847-2850.
3. C. Bénard, R. Mohammad, N. Saraswat, R. Shan, S. N. Maiti, P. G. M. Wuts, M. Stier, T. Lints, J. Bradow and J. B. Schwarz, *Synth. Commun.*, 2008, **38**, 517-524.
4. S. S. Ng, K. L. Kavanagh, M. A. McDonough, D. Butler, E. S. Pilka, B. M. R. Lienard, J. E. Bray, P. Savitsky, O. Gileadi, F. von Delft, N. R. Rose, J. Offer, J. C. Scheinost, T. Borowski, M. Sundstrom, C. J. Schofield and U. Oppermann, *Nature*, 2007, **448**, 87-91.
5. W. Kabsch, *Acta Crystallogr D*, 2010, **66**, 125-132.
6. P. R. Evans and G. N. Murshudov, *Acta Crystallogr D*, 2013, **69**, 1204-1214.
7. O. S. Smart, T. O. Womack, A. Sharff, C. Flensburg, P. Keller, W. Paciorek, C. Vonrhein and G. Bricogne, Global Phasing Ltd., 1.2.1 edn., 2011.
8. P. D. Adams, P. V. Afonine, G. Bunkoczi, V. B. Chen, I. W. Davis, N. Echols, J. J. Headd, L. W. Hung, G. J. Kapral, R. W. Grosse-Kunstleve, A. J. McCoy, N. W. Moriarty, R. Oeffner, R. J. Read, D. C. Richardson, J. S. Richardson, T. C. Terwilliger and P. H. Zwart, *Acta Crystallogr D*, 2010, **66**, 213-221.
9. P. Emsley, B. Lohkamp, W. G. Scott and K. Cowtan, *Acta Crystallogr D*, 2010, **66**, 486-501.
10. V. B. Chen, W. B. Arendall, J. J. Headd, D. A. Keedy, R. M. Immormino, G. J. Kapral, L. W. Murray, J. S. Richardson and D. C. Richardson, *Acta Crystallogr D*, 2010, **66**, 12-21.
